# Mitigation of off-target toxicity in CRISPR-Cas9 screens for essential non-coding elements

**DOI:** 10.1038/s41467-019-11955-7

**Published:** 2019-09-06

**Authors:** Josh Tycko, Michael Wainberg, Georgi K. Marinov, Oana Ursu, Gaelen T. Hess, Braeden K. Ego, Aradhana, Amy Li, Alisa Truong, Alexandro E. Trevino, Kaitlyn Spees, David Yao, Irene M. Kaplow, Peyton G. Greenside, David W. Morgens, Douglas H. Phanstiel, Michael P. Snyder, Lacramioara Bintu, William J. Greenleaf, Anshul Kundaje, Michael C. Bassik

**Affiliations:** 1 Department of Genetics, Stanford University, Stanford, CA 94305 USA; 2 Department of Computer Science, Stanford University, Stanford, CA 94305 USA; 3 Center for Personal Dynamic Regulomes, Stanford University, Stanford, CA 94305 USA; 4 Department of Bioengineering, Stanford University, Stanford, CA 94305 USA; 5 Department of Biology, Stanford University, Stanford, CA 94305 USA; 6 Program in Biomedical Informatics, Stanford University School of Medicine, Stanford, CA 94305 USA; 7 Department of Cell Biology and Physiology, University of North Carolina, Chapel Hill, NC 27599 USA; 8 Thurston Arthritis Research Center, University of North Carolina, Chapel Hill, NC 27599 USA; 9 Department of Applied Physics, Stanford University, Stanford, CA 94305 USA; 10 Chan Zuckerberg Biohub, San Francisco, CA 94158 USA; 11 Chemistry, Engineering, and Medicine for Human Health (ChEM-H), Stanford University, Stanford, CA 94305 USA

**Keywords:** Functional genomics, Genetics, CRISPR-Cas9 genome editing

## Abstract

Pooled CRISPR-Cas9 screens are a powerful method for functionally characterizing regulatory elements in the non-coding genome, but off-target effects in these experiments have not been systematically evaluated. Here, we investigate Cas9, dCas9, and CRISPRi/a off-target activity in screens for essential regulatory elements. The sgRNAs with the largest effects in genome-scale screens for essential CTCF loop anchors in K562 cells were not single guide RNAs (sgRNAs) that disrupted gene expression near the on-target CTCF anchor. Rather, these sgRNAs had high off-target activity that, while only weakly correlated with absolute off-target site number, could be predicted by the recently developed GuideScan specificity score. Screens conducted in parallel with CRISPRi/a, which do not induce double-stranded DNA breaks, revealed that a distinct set of off-targets also cause strong confounding fitness effects with these epigenome-editing tools. Promisingly, filtering of CRISPRi libraries using GuideScan specificity scores removed these confounded sgRNAs and enabled identification of essential regulatory elements.

## Abstract (web summary)

Off-target effects in CRISPR screens for essential regulatory elements have not been systematically evaluated. Here the authors find Cas9 nuclease, CRISPRi/a each have distinct off-target effects, and that these can be accurately identified and removed using the GuideScan sgRNA specificity score.

## Introduction

CRISPR-Cas9 screens^1–4^ are a powerful tool for functionally characterizing genes and non-coding cis-regulatory elements (CREs). In particular, growth screens have been employed to discover genes essential for fitness under various conditions^1,2,5,6^. CRISPR screens can also be used to interrogate the non-coding genome^7–13^. In some instances, active Cas9 nuclease is used to edit candidate functional elements (e.g., transcription factor (TF) motifs) at the sequence level by generating indels^8,13^. Alternatively, the epigenetic environment of a locus can be perturbed using nuclease-dead dCas9 fused to effector domains that can recruit chromatin silencers that modify histones with repressive marks (CRISPRi)^7,14–18^ or activators that recruit transcriptional machinery (CRISPRa)^9,17,19^.

A challenge in interpreting CRISPR screens is that Cas9 can bind to off-target genomic sites, in a manner that depends on the specificity of the sgRNA sequence^20–22^. For active Cas9, off-target activity at perfectly matched sites^23–26^ or sites with 1–2 mismatches^27,28^ has been shown to reduce cell fitness and confound gene-targeting growth screens. This reduction in cell fitness could be due to DNA damage from off-target cleavage events. Conversely, for CRISPRi/a, the impact of off-target activity on gene-targeting growth screens is thought to be minimal^3^. However, the impact of off-target activity on screens for essential non-coding regulatory elements has not been systematically studied for any perturbation.

There are reasons to expect off-target effects may be more of an issue in non-coding screens than in gene screens. For gene screens, large targetable windows are present within which all sgRNAs that induce frameshifting indels would be expected to have similar effects (i.e., a complete knockout), making the selection of highly specific sgRNAs relatively straightforward. On the other hand, screens of non-coding elements with active Cas9 often require the use of lower specificity sgRNAs because CRE components, such as TF motifs, present narrower targeting windows with fewer available sgRNAs.

Despite these challenges, CRISPR-Cas9 screens enable the systematic perturbation and characterization of candidate CREs (cCREs). A major class of cCREs that has not been functionally dissected genome-wide is CTCF sites in chromatin loop anchors. CTCF binding is enriched at the boundaries that partition interphase vertebrate genomes into TADs (Topologically Associated Domains)^29,30^; pairs of convergently oriented CTCF motifs appear to specify chromatin loop anchors^30,31^. Chromatin loops and TADs are thought to constrain enhancer-promoter interactions, adding specificity to the *cis*-regulatory wiring that connects genes with distal CREs. Deletions and inversions of CTCF sites result in reorganization of TADs^31^ and occasionally in gene expression changes^32–36^. Moreover, disruptions of CTCF occupancy have been suggested to be involved in tumorigenesis due to pathogenic rewiring of enhancer-promoter interactions^35,37–39^. Global degradation of CTCF protein showed that CTCF is required for TAD formation and maintenance and resulted in 370 differentially expressed genes after one day^40^, albeit with only small fold-changes. However, such global perturbations do not reveal the functional importance of individual CTCF sites.

To address this question, we performed a genome-wide non-coding screen for essential CTCF sites in chromatin loop anchors in the K562 leukemia cell line. We discovered that the dominant source of signal in our screen was not due to deregulated gene expression but was instead consistent with CRISPR-Cas9 off-target activity causing reductions in cell fitness, despite filtering the sgRNAs to have no perfect or 1-mismatch off-target sites. We found that the recently developed GuideScan-aggregated Cutting Frequency Determination (CFD) specificity score accurately predicted sgRNAs with confounding off-target activity and outperformed a previous score, as well as the simple number of off-target sites as a metric for identifying and removing these sgRNAs. This discovery led us to systematically explore the impact of off-target activity across different perturbations in non-coding screens. Interestingly, we observed that CRISPRi/a are similarly vulnerable to confounding off-target activity that significantly reduces cell fitness despite using non-nucleolytic dCas9. We then retrieved specificity scores for all sgRNAs in the human genome and investigated which cCREs can be reliably screened with high-specificity sgRNAs. Cas9 screens for essential functional motifs are severely limited by low availability of high-specificity sgRNAs, whereas CRISPRi/a libraries can be properly filtered to avoid confounders as their sgRNAs can be selected from a larger targeting window. Together, our results provide principles for the design and interpretation of high-throughput measurements of regulatory element essentiality.

## Results

### Genome-scale CRISPR screens for essential CTCF loop anchors

**Fig. 1:**
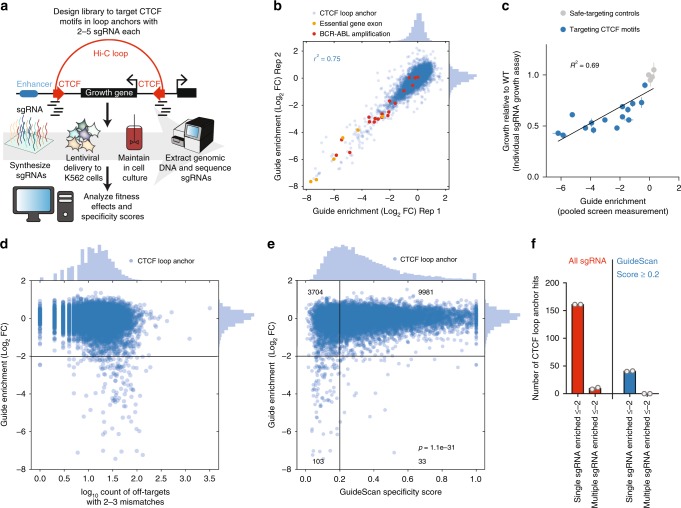
GuideScan specificity filtering of a genome-scale CRISPR-Cas9 screen for essential CTCF loop anchors. **a** Schematic of CTCF loop anchor motif screen, with 2 to 5 sgRNAs targeting each CTCF motif. **b** Fitness effects are reproducible between independently transduced biological replicates of the screen. sgRNAs targeting essential gene exons or the BCR-ABL amplification drop out during the growth screen, as expected. Guide enrichment values are the log_2_(fold-change) of an sgRNA’s sequencing counts from after the screen compared with the original plasmid pool, computed with the casTLE screen analysis software^5^. **c** The growth effects of CTCF motif-targeting sgRNA are validated in individual competitive growth assays after lentiviral delivery of single guides to K562-Cas9 cells. Error bars are standard deviation of three technical replicates. **d** Comparison of sgRNA fitness effects with the number of off-target sites with 2-3 mismatches. Any sgRNAs with off-target sites with only 0 or 1 mismatch, as determined by the GuideScan search algorithm, are excluded. **e** Low-specificity score guides are significantly enriched among CTCF motif-targeting guides with fitness effects. The Fisher’s exact test provided the p-value for the association between fitness effect and specificity using the 2 × 2 contingency table of the numbers of guides in each quadrant based on the thresholds drawn in black lines. Numbers in corners correspond to the number of CTCF site-targeting guides (blue circles) in the quadrant. The off-target search was done with GuideScan, which retrieves all off-target locations with 2 or 3 mismatches to the sgRNA spacer. sgRNAs with >1 perfect matches to the genome or >0 off-target locations with only 1 mismatch are not searchable within the GuideScan trie data structure and were excluded from this analysis. **f** Filtering for high-specificity scores removes all CTCF motifs with concordant evidence of fitness effects from multiple sgRNAs. Gray circles are screen biological replicates. Source data are available in the Source Data file

To identify essential CTCF sites, we performed a Cas9 growth screen with an sgRNA library targeting 4,022 CTCF motifs known to be at loop anchor sites in the K562 cell line according to available Hi-C and CTCF ChIP-seq evidence^30,41^ (Fig. 1a, Supplementary Data 1). The library included 2 to 5 sgRNAs per CTCF site that had an expected cleavage site within the motif. The growth effects, measured as guide enrichment from the original sgRNA library plasmid pool to the end of the screen, were highly reproducible between the two independently transduced biological replicates (*r*^2^ = 0.75, Fig. 1b). We observed strong growth effects from the internal positive control sgRNAs that target the exons of essential genes, as well as from sgRNAs targeting the BCR-ABL copy number amplification, which are expected to cause substantial toxicity due to the creation of multiple DNA double-stranded breaks^23–26^. We validated 15 individual sgRNAs using a competitive growth assay, which confirmed the growth effects observed in the pooled screen (*r*^2^ = 0.69, Fig. 1c).

To better understand the mechanistic basis for these fitness effects, we characterized the transcriptional and chromatin landscape of K562 cell lines carrying mutations induced by individual sgRNAs with validated growth effects. We chose hits where 2–3 sgRNAs targeting the same CTCF site had strong fitness effects and where changes in distal gene regulation could affect a gene that was essential in our previous Cas9 and CRISPRi/a gene screens in K562. First, we sought to confirm that sgRNAs targeting CTCF sites can disrupt CTCF binding by performing CTCF ChIP-seq on Cas9-expressing cells transduced with individual sgRNAs. Indeed, Cas9-induced indels specifically eliminated CTCF binding at the targeted CTCF, while CTCF occupancy at untargeted sites in the immediate vicinity or elsewhere in the genome remained unchanged (Supplementary Fig. 1A, B). However, a case-by-case examination of each site revealed a more complex picture. For two sites, where either only a single CTCF motif was present or the central CTCF motif relative to the ChIP-seq peak was the target of the sgRNA, we observed complete elimination of CTCF binding as expected (Supplementary Fig. 1c, right-hand side panels). In two other cases, multiple clustered CTCF motifs were present within the ChIP-seq peak; CRISPR-Cas9 perturbation specifically resulted in elimination of ChIP-seq signal over the targeted motif, as could be expected (middle panels). The last two cases (left-hand side panels) featured a site within a peak that is not strongly occupied in these K562 cells and a guide targeting a site nearby but outside the observed ChIP-seq peak, likely due to misannotation of the loop anchor motif. These last two examples naturally raised questions regarding the source of their reproducible fitness effects.

When we carried out gene expression measurements (both by qPCR and RNA-seq; Supplementary Fig. 1D–M) on cell lines carrying CTCF motif indels, we did not observe significant changes in transcript levels for genes located in the genomic neighborhood of the targeted CTCF sites. Similarly, ATAC-seq experiments did not reveal significant changes in chromatin accessibility (Supplementary Fig. 1N). Altogether, these experiments did not nominate changes in gene expression or chromatin structure near the CTCF motifs as likely causes of the observed growth effects for any of the motifs we aimed to validate. Instead, we wondered whether off-target activity could explain these results, since off-target effects have previously been found to generate confounding signal in CRISPR-Cas9 growth screens^23–25,27,28^ and the sgRNA fitness effects in our screen were weakly correlated with their number of predicted off-target sites in the human genome (Pearson’s *r* = 0.13, Fig. 1d).

### Specificity model reveals confounder in CTCF screens

To further explore the possibility that off-target activity was responsible for the screen results, despite library filtering, we retrieved specificity scores^42^ for every sgRNA. These sgRNA-level aggregate scores are determined by (1) searching reference genomes for off-target binding locations, (2) predicting the Cas9 activity across those sites given the pattern of mismatches between the sgRNA and the genomic DNA, and (3) aggregating these predicted Cas9 activities into a final score. Different implementations of this workflow have resulted in a variety of software tools providing specificity scores^20,42–46^. We found that aggregated CFD specificity scores from GuideScan^42^ correlate very well with data from Guide-seq^21^, an unbiased off-target measurement assay for Cas9 (Spearman’s ρ = −0.84, Supplementary Fig. 2A). These GuideScan scores outperformed MIT aggregate specificity scores^20^ (Supplementary Fig. 2B). Notably, the selected sgRNAs that conferred reproducible fitness effects without affecting nearby essential gene expression had moderate MIT specificity scores ranging from 20–54 (mean = 34) but very low GuideScan scores ranging from 0–0.24 (mean = 0.06). GuideScan scores are a weighted function of all off-target locations with 2 or 3 mismatches to the sgRNA spacer that considers the position, number, and nucleotide identity of the mismatches. Importantly, this analysis focuses on further refinement of reasonably designed sgRNAs, as all very low-specificity sgRNAs with >1 perfect match in the genome or any off-target locations with only 1 mismatch had already been excluded.

In the full screen data, we observed a striking bias for low specificity scores among the sgRNAs that confer large fitness effects (*p* = 1.1e−31, Fisher’s exact test, Fig. 1e). Indeed, the majority (76%) of CTCF motif-targeting sgRNAs that have guide-level log_2_(fold-change) ≤ −2 also had GuideScan specificity scores ≤0.2 (on a scale of 0 to 1, where 0 indicates least specificity or greatest off-target activity), representing an 8.4-fold odds ratio. In the case of our CTCF screen, 4% of CTCF loop anchors had strong evidence of essentiality (Guide enrichment log_2_(fold-change) ≤ −2) with a single sgRNA, but only 0.2% had such evidence from multiple sgRNAs (Fig. 1f). This disparity is unexpected given that the sgRNAs targeting the same site should have similar effects but is consistent with the sgRNAs having different off-target effects. After filtering for high-specificity sgRNAs with the GuideScan score, the number of CTCF loop anchors with evidence of essentiality from multiple sgRNAs dropped to zero (out of 2968 motifs targeted with multiple high-specificity sgRNAs). Together, these results experimentally validated the new GuideScan specificity score as an effective predictor of off-target activity and a more useful parameter for screen filtering than the absolute number of off-target sites or a previous aggregate specificity score.

### Dense-tiling CTCF loop anchors with pooled Cas9 screens

**Fig. 2:**
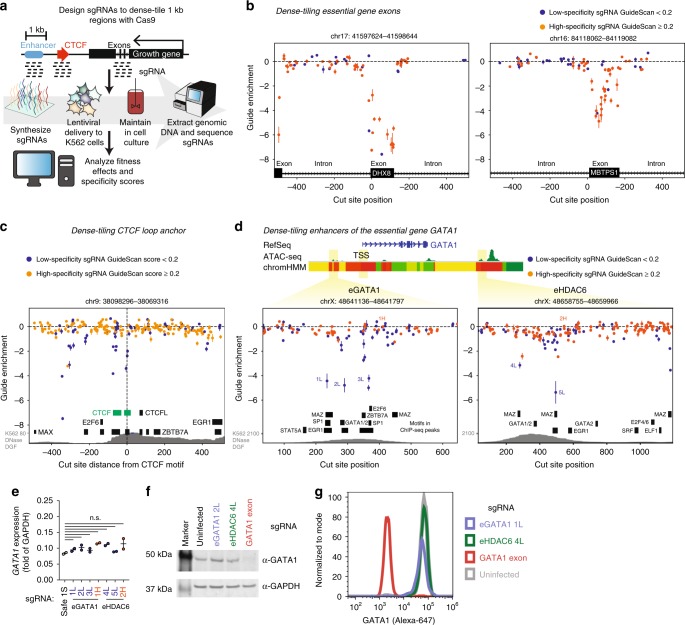
Low-specificity sgRNAs confound identification of essential motifs in dense-tiling screen of loop anchors and enhancers. **a** A dense-tiling Cas9 growth screen was performed with sgRNAs densely tiling two types of regions: (1) 1 kb windows around select hit and non-hit CTCF loop anchors from the CTCF motif screen and (2) two enhancers of *GATA1*, previously called eGATA1 and eHDAC6. **b** As a positive control, we verified that the dense-tiling screen correctly maps the boundaries of exons of essential genes with high-specificity sgRNAs. Each point is the average enrichment of two biological replicates and the bar is the standard error. **c** Dense-tiling screen results from a 1 kb region centered on a motif that was a false positive hit in the original motif-targeting screen (targeted with sgRNAs 15776 and 15777 and also shown in Fig. 1 and Supplementary Fig. 1). All evidence for the essentiality of a CTCF motif comes from low-specificity sgRNAs. Motifs in ChIP-seq peaks are shown as black boxes and CTCF motifs as green boxes. **d** Dense-tiling screen results from two regions containing enhancers of the essential gene *GATA1*. sgRNAs selected for validation studies are labeled (e.g., 1 L represents the first sgRNA with a low specificity score). ChromHMM is colored according to the 15-state scheme^76^ (briefly, reds are predicted promoter states, yellows are enhancer states, and greens are other transcriptionally active states). **e** The enhancer motif-targeting sgRNAs identified in **d** do not significantly decrease GATA1 expression according to qPCR (*p* > 0.05, ANOVA). Each dot is a sgRNA infection biological replicate. **f** The sgRNAs identified in **d** do not significantly decrease GATA1 protein expression according to Western blot. **g** The sgRNAs identified in **d** do not significantly decrease GATA1 protein expression according to flow cytometry for GATA1 protein level. Additional validation data are shown in Supplementary Fig. 4. Source data are available in the Source Data file

To further test whether off-target activity could explain the hits from the CTCF motif screen, we designed a dense-tiling sgRNA library targeting 270 CTCF sites, including full tiling of each such site (all possible sgRNAs within 1 kb), using up to 400 sgRNAs per site (Fig. 2a). We chose CTCF sites from four categories: hits called by casTLE analysis before filtering with GuideScan scores, the Hi-C loop partners of these hits, non-hits, and the loop partners of the non-hits (see Methods section). We expected three possible results from densely tiling the loop anchors: (1) truly essential CTCF motifs would result in a strong peak of signal from high-specificity sgRNAs that generate indels near the motif (i.e., +/−20 bp), (2) regions that were essential for reasons distinct from the CTCF motif, such as being copy number amplified^23,25,26,47^, would result in uniformly strong growth effects from both low-specificity and high-specificity sgRNAs irrespective of whether the sgRNAs overlap the motifs, and (3) non-functional motifs would only have strong signal from low-specificity sgRNAs, if any. This dense-tiling screen was performed at high coverage (~12,000 cells per sgRNA) and yielded highly reproducible guide effect measurements (*r*^2^ = 0.92, Supplementary Fig. 3A). As expected, positive control sgRNAs targeting ten essential genes were strongly depleted (Supplementary Fig. 3B). We observed uniform depletion of high-specificity and low-specificity sgRNAs tiling regions near the *BCR-ABL* amplification but not elsewhere (Supplementary Fig. 3C, D), as expected. Both high-specificity and low-specificity sgRNAs had strong growth effects when targeting exons of essential genes but no effect in the neighboring introns (Fig. 2b), demonstrating that the dense-tiling screen can discern the short functionally relevant sequences of coding exons from background with high fidelity. Strikingly, the great majority (93%) of sgRNAs tiled within the 1 kb CTCF loop anchor regions that had a strong fitness effect were, again, low-specificity guides with GuideScan scores ≤0.2 (*p* = 2.3e−233, Fisher’s exact test, Supplementary Fig. 3E). While the previous motif-targeting library only used 2–5 sgRNAs per motif, this dense-tiling library included all possible guides overlapping a window of +/−20 bp of the CTCF motif centers. Despite this increase in sgRNA density, after filtering with GuideScan scores, we still found zero CTCF motifs with evidence of essentiality from multiple high-specificity sgRNAs (Fig. 2c and Supplementary Fig. 3F, G). We therefore concluded that the observed hits in the CTCF screens were consistent with off-target activity. This result suggests (but does not conclusively prove) that the CTCF loop anchors we tested in K562 are not essential for cell growth in normal conditions, which appears consistent with recent observations that degron-mediated depletion of loop anchor proteins can have minimal effects on transcription^40,48–51^. Notably, functional redundancy of CTCF sites or inefficient genome editing could also lead to false negatives. While we could not fully explain why no CTCF sites were convincing hits in these screens, we consistently found strong evidence that GuideScan scores reveal confounding off-target activity and set out to explore the utility of this approach on other non-coding CRISPR screens.

### Off-target activity in Cas9 screens of enhancers

To test our ability to dissect the essentiality of non-coding elements beyond chromatin loop anchors, we also densely tiled two enhancers which regulate expression of the essential gene *GATA1* in K562 cells, with 110 and 174 sgRNAs to span the entire 611 bp and 1.1 kb regions, respectively. These enhancers, named eGATA1 and eHDAC6, were previously identified in a CRISPRi tiling growth screen in K562^7^, but their constituent functional motifs remain uncharacterized. We sought to identify these with higher resolution dissection by Cas9 dense-tiling. These screens revealed narrow peaks defined by 1–2 sgRNAs that overlapped known TF ChIP-Seq motifs within the DNase hypersensitive sites in the enhancers^41^ (Fig. 2d). However, these sgRNAs were again of low specificity, raising doubts that their targets were in fact essential motifs and motivating a careful validation of the sgRNAs and their effects on *GATA1* expression. We installed the sgRNAs individually into K562 cells and found that this resulted in indel mutations (37–98%) in the genomic DNA at the corresponding target motifs (Supplementary Fig. 4A). These sgRNAs also caused significant growth phenotypes (Supplementary Fig. 4B) which correlated with the growth effects measured in the pooled screen (*r*^2^ = 0.76, Supplementary Fig. 4C). However, there were no concordant changes in *GATA1* expression as measured by qPCR, Western blot, or flow cytometry (Fig. 2e–g and Supplementary Fig. 4D). These experiments demonstrate that even sgRNAs targeting TF motifs in bona fide enhancers can have reproducible growth screen effects that are unrelated to the expression of their nearby essential gene, and that the GuideScan specificity score is useful to help identify such confounded sgRNAs. Further, these results suggest that even dense-tiling can potentially miss critical motifs or, more interestingly, that no single sgRNA might be sufficient to disrupt the activities of these enhancers.

### CRISPRi/a off-target activity causes large fitness effects

**Fig. 3:**
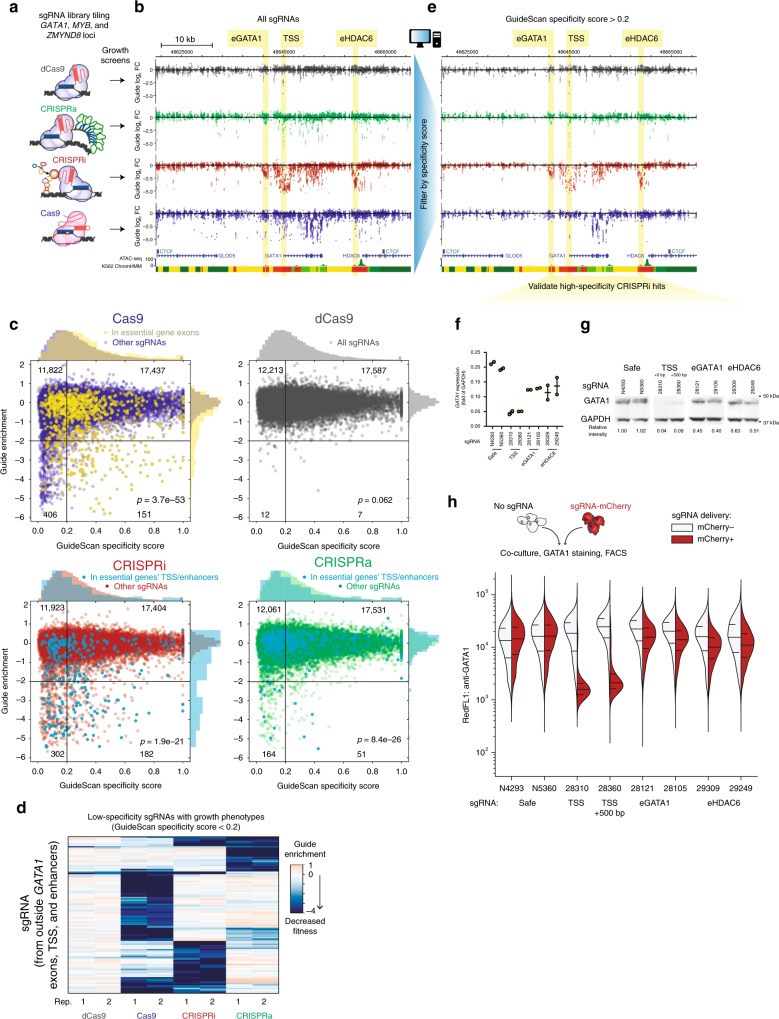
GuideScan specificity filtering of CRISPRi library reduces false positives. **a** Four parallel screens were conducted tiling the loci of essential growth genes *GATA1, MYB*, and *ZMYND8* using the four platforms Cas9, CRISPRa, CRISPRi and dCas9. **b** Zoomed-in view of screen data around essential gene *GATA1*. Highlighted are regulatory elements with known effects on cell growth: enhancers eGATA1 and eHDAC6, and the *GATA1* transcription start site. ChromHMM is colored according to the 15-state scheme^76^ (briefly, reds are predicted promoter states, yellows are enhancer states, and greens are other transcriptionally active states). Each point is the average enrichment of two screen biological replicates and the bar is the standard error. **c** Enrichment of growth effects among low-scoring sgRNAs with no perfectly matching and no 1-mismatch off-target sites. *p*-value from the Fisher’s exact test for the 2 × 2 table with quadrants as drawn and guide counts as labeled in the corners; these counts include all the sgRNAs regardless of the categories indicated in colors. **d** Clustering of low-specificity sgRNAs reveals that each perturbation has off-target activity that reduces cell fitness with a unique subset of the low-specificity sgRNAs. Shown are the subset of sgRNAs that are upstream of eGATA1 or downstream of eHDAC6 (i.e., sgRNAs with predominantly off-target effects) and that also have a strong guide enrichment ≤ −3 in at least one replicate. Color scale is the log2 fold-change guide enrichment. **e** Filtering of sgRNAs in panel B with GuideScan specificity scores reduces noise. **f** After filtering, the CRISPRi sgRNAs in peaks have validated effects on *GATA1* expression by qPCR (*p* < 0.05, ANOVA). Each dot is a sgRNA infection biological replicate. **g** Effects of indicated sgRNAs on *GATA1* protein expression measured by Western blot. **h** Effects of indicated sgRNAs on *GATA1* protein expression measured by flow cytometry. Here, cells expressing an sgRNA and mCherry were co-cultured with the blank parental cell line, stained for GATA1 protein, and analyzed by flow cytometry. We then compared the distribution of GATA1 protein level between the mCherry + and blank control cells from the same sample. Horizontal lines show the median and quartiles. Source data are available in the Source Data file

CRISPRi and CRISPRa have also been used to screen for functional non-coding elements, but the potentially confounding effect of off-target activity with these platforms in the context of non-coding essential regulatory elements has not been studied. To systematically compare these technologies, we performed a tiling screen around three essential genes in K562 cells (*GATA1, MYB*, and *ZMYND8*); the library consisted of a total of 32,791 sgRNAs targeting a total of 794 kb including candidate regulatory elements, annotated exons and intervening genomic space. We screened this library with four different CRISPR-Cas9 platforms: active Cas9, nuclease-dead dCas9, CRISPRi (dCas9-KRAB^17^), and CRISPRa (dCas9-SunTag-VP64^52^) (Fig. 3a). As expected, in the active Cas9 screen we observed strong negative fitness effects for sgRNAs targeting exons, and in the CRISPRi screen we observe strong signals for sgRNAs targeting known essential enhancers and promoters^7,53^ (Fig. 3b and Supplementary Fig. 5A–D). We also found that for CRISPRa and dCas9 screens, sgRNAs that targeted transcriptional start sites (TSS) of essential genes exhibit negative fitness effects (Fig. 3b and Supplementary Fig. 5D); for dCas9, this observation may be due to the binding of dCas9 interfering with the transcriptional initiation machinery^17,54^.

However, for each screening modality we also noticed sgRNAs with strong negative fitness effects that did not target candidate regulatory elements or annotated coding sequences and for which neighboring sgRNAs did not exhibit concordant effects (Fig. 3b). Again, we suspected that the growth effects of these guides might be due to off-target activity and used GuideScan aggregate specificity scores in order to investigate this possibility. Indeed, we observed a striking enrichment for low-specificity sgRNAs among the set of sgRNAs with strong negative fitness effects in the Cas9, CRISPRi, and CRISPRa screens (*p* < 1.9e−21 for all, Fisher’s exact test, Fig. 3c). We questioned whether the sets of sgRNAs with putative off-target activity were highly overlapping between each CRISPR-Cas9 platform. Strikingly, this was not what we observed. In fact, sets of low-specificity sgRNAs that show significant fitness effects with Cas9, CRISPRi, or CRISPRa are largely non-overlapping (Fig. 3d), suggesting the off-target effects are specific to each CRISPR-Cas9 platform. Thus, off-target growth effects appear to be a function of both the sites targeted by an sgRNA and the mode of perturbation.

We questioned whether these off-target growth effects were purely a function of the absolute number of off-target sites or specific to a subset of off-target sites. We and others have shown that, in the context of coding gene screens, the number of perfect matches or 1-mismatch off-targets correlates with growth phenotypes^27,28^. However, the analyses presented here do not include any sgRNAs with perfect genomic matches at any other place in the genome, nor sgRNAs with 1-mismatch off-targets. Across all four CRISPR-Cas9 platforms used in the tiling screens, the GuideScan score was predictive of off-target effects on cell fitness (Fig. 3c and Supplementary Fig. 6A), yet there was very weak correlation between growth effects and the absolute number of off-target sites (with 2 or 3 mismatches each), especially for CRISPRi/a (Supplementary Fig. 6B, C). Indeed, some outlier sgRNAs with thousands of off-target sites had no effects on growth. Thus, when designing and interpreting screens, the propensity to bind or cut as captured by the specificity score should be considered, rather than simply the number of off-target binding locations. These propensities are predicted for each off-target location by the CFD score^44^ as a weighted function of the mismatch number, position, and nucleotide identity, and then aggregated across all off-target locations into a GuideScan aggregate specificity score. Lastly, the optimal GuideScan score cutoff for filtering out false positives while retaining library density varies slightly but is approximately 0.2 for CRISPRi/a and Cas9 (Supplementary Fig. 6D).

### High-specificity CRISPRi libraries identify essential CREs

While the appearance of confounding off-target activity in CRISPRi screens was unexpected, GuideScan scores proved useful to identify confounded sgRNAs. We next asked if the removal of low-specificity sgRNAs would improve the reliable identification of expected regulatory elements (e.g., the TSS and the two enhancers of *GATA1*). We thus filtered out guides with GuideScan scores ≤ 0.2, which did indeed remove confounded sgRNAs while preserving strong CRISPRi signal at these enhancers and promoters (highlighted regions in Fig. 3e).

To confirm that these high-specificity sgRNAs in peaks had bona fide effects on the expression of *GATA1*, we delivered single guides by lentivirus and measured *GATA1* expression by qPCR and Western blot (Fig. 3f, g). Whereas targeting the *GATA1* TSS or a CRISPRi peak 500 bp downstream of the TSS both resulted in near-complete knockdown (to 4–9% of protein levels in the control cells), the enhancer-targeting sgRNAs provided partial knockdown (to 40–63% of control protein levels), and expression levels were highly correlated between RNA-level qPCR and protein-level Western blot (*R*^2^ = 0.92, Supplementary Fig. 7A). Flow cytometry for GATA1 protein levels confirmed that CRISPRi enhancer repression resulted in partial knockdown across the population of cells, as opposed to complete silencing observed when targeting the TSS (Fig. 3h). Together, these experiments validated that the high-specificity sgRNAs from the tiling CRISPRi screen resulted in on-target repression of the expected essential gene.

We next wondered if off-target activity might confound other CRISPRi/a non-coding growth screens for other types of elements. To directly compare the different CRISPR-Cas9 platforms with a shared library of sgRNAs, we performed parallel screens with our CTCF motif-targeting sgRNA library in K562 using CRISPRi, CRISPRa, dCas9, and Cas9 (Supplementary Fig. 8A–C). When we analyzed the specificity scores of this library, we found that these CRISPRi and CRISPRa screens again showed a significant bias towards low-specificity sgRNAs having strong growth effects (Supplementary Fig. 8D). The Cas9 screen in this experiment was maintained with lower coverage (cells per sgRNA) and was thus noisier than the Cas9 screen in Fig. 1; interestingly, we found that this enrichment for low-specificity sgRNAs was less pronounced but remained highly significant (*p* = 1.1e−9, Fisher’s exact test), showing that the signature of off-target effects can be disguised in noisy screens. As with our tiling library, we found that the sets of low-specificity sgRNAs that show significant fitness effects with Cas9, CRISPRi, or CRISPRa are largely non-overlapping, reproducing the previous observation that off-target effects are specific to each CRISPR-Cas9 perturbation (Supplementary Fig. 8E). Again, the CRISPRi/a growth phenotypes were not reproduced when employing dCas9 with the same sgRNAs, demonstrating these off-target effects are not due to dCas9 binding alone.

To investigate the generality of these CRISPRi off-target growth effects across cell types, we retrieved GuideScan specificity scores for guide libraries from published screens targeting the promoters of genes with dCas9-KRAB-MeCP2 in SH-SY5Y and HAP1 cells^18^. These screens found reproducible, validated hits, but also found that some sgRNAs targeting known non-essential genes had unexpected growth effects. Here, we found that these sgRNAs also had lower specificity scores (Supplementary Fig. 9A). These results suggest that using CRISPRi with low-specificity sgRNAs can be associated with strong fitness effects in other cell types. Similarly, we found evidence that low-specificity sgRNAs targeting Cas9 near the TSS of genes were also enriched for fitness effects in several other cell types in previously published screens (Supplementary Fig. 9B). Together, these results suggest that our findings can be generally useful for filtering and interpreting growth screens, regardless of the cell type used.

### Impact of low-specificity sgRNAs on non-coding screen design

**Fig. 4:**
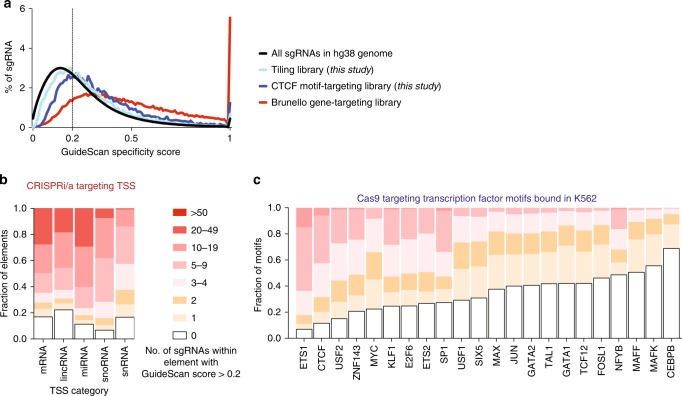
High-specificity CRISPR-Cas9 screen designs for non-coding elements. **a** Distribution of GuideScan specificity scores for two non-coding libraries from this study and a gene-targeting library, in comparison to all possible sgRNA. **b** Most TSSs can be targeted with multiple high-specificity sgRNA. Fraction of TSS in the ENCODE SCREEN database of ccREs that can be targeted with dCas9-based epigenome editors within a window of +/−100 bp, after filtering for GuideScan scores >0.2. **c** Fraction of motifs in TFBS motifs that can be targeted with sgRNAs with a cut site in the motif, after filtering out low-specificity sgRNAs

Finally, we investigated the extent to which non-coding elements can be targeted with high-specificity sgRNA libraries. To address this question, we characterized the distribution of GuideScan specificity scores for a number of possible screen designs. We observed that our tiling screen and CTCF site screen libraries contained significantly more low-specificity sgRNAs than Brunello^44^, a genome-wide coding gene-targeting library (*p* < 0.0001, Mann–Whitney test, Fig. 4a), reflecting the inherently poorer specificity of sgRNA libraries that densely tile regions or target relatively small motifs. We then designed libraries targeting all candidate cis-regulatory elements (or ccREs) which were identified in the ENCODE SCREEN databases^55,56^. At the time of our analysis, the SCREEN databases contained 1.31 million individual ccREs, with a median length over 200 bp (Supplementary Fig. 10A). We specifically focused on CRISPRi/a epigenetic perturbation designs and imposed a minimum requirement of including at least 5 sgRNAs of sufficiently high specificity for each element (to enable robust statistical analyses of functional effects at the element level). We find that 89% of SCREEN cCREs can be targeted with ≥5 sgRNAs at a GuideScan cutoff of 0.2 (Supplementary Fig. 10B) although this varies by type of target element. For example, we find that 62% of human lncRNA TSS elements can be targeted with ≥5 CRISPRi sgRNAs with a specificity score >0.2, even when selecting sgRNAs from a conservative window of only +/−100 bp from the TSS (Fig. 4b). Overall, most ccREs can be targeted with epigenome editing tools even after filtering the sgRNAs that are most likely to be confounded by off-target effects.

However, most ccREs are composed of multiple regulatory units, such as transcription factor binding sites (TFBSs), and achieving mechanistic understanding of cCRE function will require perturbing these regulatory units, individually or in combination. To assess the ability of Cas9 to enable more fine-grained regulatory element mapping, we designed motif-level screens for 27 different human TFs targeting all of their annotated and occupied motifs in K562 cells and summarized the specificity score distributions for each. We find that guide specificity filtering restricts the ability to target TF motifs to a varying extent for different TFs: for example, only 31% of CEBPB motifs can be targeted with even a single overlapping sgRNA at a GuideScan cutoff of 0.2 (Fig. 4c), whereas for TFs such as ETS1, 64% motifs can be targeted with 5 or more such guides. Taken as a whole, Cas9 TF motif screens, as well as splice site screens (Supplementary Fig. 10C), are subject to more limiting design restrictions than screens targeting cCREs with CRISPRi/a, because the sgRNAs for these Cas9 non-coding screens must overlap the narrow target element directly while sgRNAs for CRISPRi/a cCRE screens can be selected from a larger targeting window. These designs provide a guideline for focusing future screens for essential regulatory elements on the motifs and cCREs that can be targeted with high-specificity guides, and we provide scripts here to both aid in the analysis of previous libraries for specificity, as well as the design of new sgRNA libraries for non-coding elements with greater specificity.

## Discussion

Here, we found that off-target activity confounds Cas9, CRISPRi, and CRISPRa screens for essential regulatory elements in K562 cells by conducting several screens using sgRNA libraries designed to edit motifs and tile regions of interest in an unbiased fashion. Notably, these sgRNAs had already been filtered to lack 0–1 mismatch off-target sites; i.e., this confounding activity was found in sgRNAs with only 2+ mismatch off-target sites, which may have passed previous design requirements. Importantly, use of GuideScan aggregate specificity scores to identify sgRNAs with only 2+ mismatch off-targets and their propensity to mediate Cas9 binding/cutting could resolve most of these issues. We present a strategy and software to use this score to filter screens for essential non-coding elements.

Surprisingly, we find that low-specificity sgRNAs are the dominant confounding factor not only for active Cas9 screens but also for dCas9-mediated perturbations such as CRISPRi and CRISPRa. Cas9 generates double-strand breaks (DSB), so a large number of off-targets for a given sgRNA could result in a major fitness effect due to cellular toxicity as a result of activation of the DNA damage response and apoptosis^23,25–27,53^, regardless of the location of off-target sites. In contrast, dCas9-recruited epigenetic perturbations do not generate DSBs, and their off-target effects are expected to be location-dependent. Interestingly, these off-target effects cannot be fully accounted for by dCas9 binding itself, as we tested the same sgRNAs with all four CRISPR-Cas9 platforms, and nearly all sgRNAs showed unmeasurable growth effects with dCas9 alone. Future studies of the mechanisms of CRISPRi/a off-target toxicity will improve our understanding of the cellular response to these perturbations and enable improved experimental designs. This is especially relevant for non-coding screens, which may be particularly vulnerable to confounding off-target activity given the need to target small regions with few available sgRNAs. As an example of the impact that off-target effects can have, growth screens targeting CTCF sites in K562 cells returned only hits that on closer examination were confounded by off-target activity. None of the CTCF sites that we characterized in more detail in cell lines expressing sgRNAs had a measurable impact on gene expression or chromatin states in the genomic neighborhood (Supplementary Fig. 1). Dense-tiling of those motifs also did not find concordant evidence of CTCF site essentiality from multiple high-specificity sgRNAs, which further supports the conclusion that the hits were false positives. Although this is unexpected, it is potentially consistent with recent studies that reported acute global degradation of either all CTCF protein^40^ or all of the loop anchor cohesin component RAD21 in cells^49^ did not result in dramatic changes in gene expression. Individual CTCF site deletions at the boundaries of TADs containing developmental genes were recently reported to have no effect on nearby gene expression or developmental phenotypes in mouse embryos^48,50^. Therefore, our results appear consistent with other evidence that individual CTCF sites are dispensable for gene regulation in many contexts.

However, our CTCF screen data could also include false negatives; it remains possible that some of the loop anchor CTCF motifs we targeted may be functional but redundant, or that CTCF sites with the greatest functional relevance under standard growth conditions may not actually be at loop anchors or may be at locations we did not target efficiently with multiple sgRNAs. While the targeted loop anchors were called from K562 Hi-C data, it remains possible that the structural variation of the K562 genome^57^ leads to lowered CTCF site targeting accuracy or lower efficiency of disrupting all copies of a CTCF site and thus more false negatives than would appear in a CTCF site screen in a different cell type. In terminally differentiated cells, such as K562, chromatin states may not be dramatically disrupted by the absence of an individual loop anchor CTCF site. While we cannot conclusively explain the absence of essential CTCF sites in our data, the off-target driven false positive CTCF sites exemplified how off-target activity poses a particular challenge to CRISPR screens for essential non-coding elements.

Our findings have implications for the design and analysis of future screens. Given that (1) validation experiments of individual screen hits are time-intensive and low-throughput, and (2) there is a growing interest in global analyses of aggregated non-coding screen data, computational models for filtering out low-specificity sgRNAs are crucial to identify bona fide hits and to diagnose systemic problems before data aggregation. We find that off-target effects on cell fitness are not predictable solely from the absolute number of off-target sites for these sgRNAs, although that simple metric is often used when designing and ranking sgRNAs. In contrast, we find that the data-driven GuideScan specificity score, which accounts for the position and type of mismatches to provide a weighted assessment of Cas9’s affinity for each potential off-target site, provides a more accurate determination of off-target potential. While the GuideScan off-target search algorithm has previously been described^42^, the GuideScan aggregate specificity score (i.e., aggregating CFD specificity scores across GuideScan’s list of off-target sites) was not reported in the literature. We found a striking correlation of this score with fitness effects in non-coding screens, and also with direct measurements of off-target cutting using Guide-seq, which exceed previous scores and suggest the use of this score to filter non-coding CRISPR screens will be broadly useful.

We find that targeting a substantial fraction of individual TFBSs with high-specificity sgRNAs when using Cas9 is often impossible, although this fraction varies widely between different TFs. This constraint imposes a significant limitation on Cas9 growth screens directed at elements as small as TFBSs (<30 bp). On the other hand, at the level of an individual cCRE (>150 bp), sufficiently many high-specificity sgRNAs can generally be found for CRISPRi and CRISPRa screens. Notably, coding gene screens also benefit from larger available sequence from which to choose sgRNAs.

However, GuideScan models only the potential extent of off-target cleavage activity and very frequently gives low specificity scores for sgRNAs that have no effect on the phenotypic outcome of cell growth. One exciting future direction suggested by our study is the development of models to predict the phenotypic consequence of off-target activity, which can now be enabled by high-throughput datasets such as these. By integrating features including the chromatin state of off-target binding locations and the essentiality of genes near those off-target locations, it may be possible to tailor models to predict which particular sgRNAs would be confounded if used with each CRISPR-Cas9 platform.

We expect that the impact of low-specificity guides is dependent on the phenotype being screened. Low-specificity sgRNAs have a greater potential to confound growth screens, likely because proliferation is affected by many factors in the cell, while screens employing different selection strategies may be less sensitive to these effects. Studies of cCRE effects that involve measuring the RNA or protein products of cognate genes, separating cell populations according to expression levels, and then identifying the particular sgRNAs associated with each expression level may also be less affected by off-target effects. Similarly, experiments that couple CRISPR-Cas9 screens to single-cell readouts of gene expression^58–60^ or chromatin accessibility^61^ may likewise overcome limitations associated with growth as a readout.

Regardless, limitations remain that will be best addressed by the development of perturbation systems that either expand the targetable sequence space or minimize off-targets. Efforts in both of these directions are ongoing, e.g., devising guide design strategies that reduce off-target effects such as truncated guides^27,62^, engineering high-specificity variants of Cas9^63,64^, and exploring the possibilities for adapting other CRISPR enzymes without strict PAM requirements^65–68^. We expect that the combination of technological improvements, judicious screen design, and careful data analysis that explicitly considers guide specificity will enable the comprehensive functional characterization of the essential regulatory elements in the human genome.

## Materials and methods

### Cell lines and cell culture

All experiments presented here were carried out in K562 cells (ATCC CCL-243)^5^. Cells were cultured in a controlled humidified incubator at 37 °C and 5% CO_2_ in RPMI 1640 (Gibco) media supplemented with 10% FBS (Hyclone), penicillin (10,000 I.U./mL), streptomycin (10,000 µg/mL), and L-glutamine (2 mM). Experiments were performed in four modified K562 cell lines: K562 stably expressing SFFV-Cas9-BFP, K562 expressing SFFV-dCas9-BFP, K562 expressing dCas9-SunTag-VP64^3^ (CRISPRa), and K562 expressing SFFV-dCas9-KRAB-BFP (CRISPRi). The CRISPRa cell line expressing the SunTag system was a gift from the lab of Jonathan Weissman.

### CTCF motif-targeting sgRNA library design

We selected CTCF motifs in loop anchors to target as follows. We started with 6057 loops present in K562 cells and focused on the 4,892 loop anchors that had previously annotated motifs overlapping ChIP-seq peaks^30^ for CTCF (using STORM^69^), such that the CTCF motifs were convergently oriented into the loop, which is suggested to be the correct orientation for loop formation. We further restricted to 4172 loop anchor CTCF motifs that could be targeted with with at least two sgRNAs per site, as defined by our guide filtering criteria below. Some of these CTCF motif targets were in exons of genes or near the BCR-ABL amplification, which could result in growth effects unrelated to CTCF binding, so they were treated separately during analysis, resulting in a final count of 4022 Type 0 CTCF loop anchor motifs. Finally, a set of control sgRNAs targeting safe regions was added. Briefly, safe-targeting negative control sgRNAs are highly filtered to target a non-functional genomic site and avoid having severe growth effects while controlling for the effect of inducing a double strand break^27^. An additional 310 CTCF and Rad21 sites (Types 1–5) were selected with alternative methods (Supplementary Materials and Methods) and also targeted with sgRNAs in the library, but these were filtered out during analysis and not included in Fig. 1 for the sake of clarity and because this small alternative set was similarly confounded by off-target activity and lacking hits. For sites that passed our filtering criteria, we selected a maximum of 5 sgRNAs per site. 95% of these sgRNAs overlapped a K562 CTCF ChIP-seq peak in our CTCF ChIP-seq data.

To minimize off-target effects, we filtered out sgRNAs that had exact or 1-mismatch off-target instances within another CTCF site or inside exons of GENCODEv19^70^ genes, to avoid confounding activity from targeting multiple CTCF sites or knocking out genes. We also filtered out guides with >2 0-mismatch, >10 1-mismatch, >50 2-mismatch, or >200 3-mismatch genome-wide off-targets. We defined off-target matches by aligning the guides to the hg19 version of the human genome using BWA ‘aln’ with the flags -N -n 4 -o 0 -k 0 -l 7^71^. However, the screen data presented in Fig. 1 and Supplementary Fig. 8 is further filtered more stringently to only display sgRNAs with no perfectly matching and no 1-mismatch off-target sites as defined by the GuideScan search algorithm. We also filtered out guides with too low (<20%) or too high (>80%) GC content and guides containing confounding oligonucleotides that might affect the expression of the guide or PCR steps, where confounding oligonucleotides are defined as those that either end in GGGGG, contain TTTT, or contain restriction cut sites (CTGCAG, GAAGAC, GTCTTC, CCANNNNNNTGG, GCTNAGC).

### CTCF sgRNA screen execution

Oligonucleotide libraries (Supplementary Data 1) were synthesized by Agilent and then cloned into an sgRNA expression vector pMCB320 (Supplementary Table 1) that had been cut with BstXI and BlpI restriction enzymes, by ligation with T4 ligase (NEB M0202M). To generate sufficient lentivirus to infect the library into K562 cells, we plated 293 T cells on 15-cm tissue culture plates. Two hundred and ninety three T cells were transfected with third-generation packaging plasmids and sgRNA-encoding vectors. After 48 h and 72 h of incubation, lentivirus was harvested. We filtered the pooled lentivirus through a 0.45-μm PVDF filter (Millipore) to remove any cellular debris. K562 cells were infected with our lentiviral sgRNA library. Infected cells grew for 3 days before the cells were selected with puromycin (0.7 μg/mL, Sigma). After 3 days of selection, infection efficiency was monitored using flow cytometry (BD Accuri C6). Once the cells reached 90–100% mCherry + cells, they were spun out of selection and allowed to recover in normal RPMI 1640 media. Cells were then maintained at 3000× coverage (cells per sgRNA). Cells were maintained in log growth conditions each day by diluting cell concentrations back to a 0.5 × 10^6^ cells/mL. These conditions were used for the Cas9, dCas9, CRISPRi, and CRISPRa screens performed with this library. After 14 days of growth, cells were spun down (300 × *g* for 5 min). Genomic DNA was extracted with Qiagen’s Blood Maxi Kit, and the sgRNA library composition was sequenced and compared to the plasmid library using casTLE^5^ version 1.0 (available at https://bitbucket.org/dmorgens/castle).

The screen was repeated in K562-Cas9 cells at 11,000× maintenance coverage for 23 days, starting from a frozen aliquot of cells after library transfection and puromycin selection (frozen at day 6). After the screen, genomic DNA was harvested and sgRNAs were amplified and sequenced. The high-coverage screen showed better reproducibility between biological replicates (Supplementary Fig. 8C) and was used for all analyses shown in the main text (Fig. 1).

### Dense-tiling screen library design

The dense-tiling screen employed densely tiled sgRNAs in short 1 kb windows around CTCF motifs, enhancers, and exons of essential genes. First, we densely tiled the regions around the CTCF motif screen hits as identified by casTLE (see below), a GC-matched set of regions around non-hit CTCFs, and the loop partner CTCFs that looped to any of these positive or negative CTCFs in a K562 Hi-C dataset^30^. Non-hit CTCFs were selected from the set of CTCF sites with enrichment magnitudes less than 0.5 for all guides in all motif-targeting Cas9, CRISPRi/a, and dCas9 screens. We selected all sgRNAs provided by the GuideScan design tool within the CTCF motif and up to 500 bp on each side, for a total of 1020 bp. For each CTCF hit, we selected a 1020-bp region around a ‘GC-matched’ non-hit CTCF with a GC content within 5% of the GC content of the 1020-bp region around the CTCF hit. In addition, we densely tiled the essential enhancers eGATA1 and eHDAC6 as positive controls and added 1000 safe-targeting guides as negative controls. As an additional positive control, we included all guides from a 10-guide gene-targeting library^27^ for the essential genes *CTCF*, *RAD21*, *SMC1A*, *SMC3*, *MYC*, *GATA1*, *MYB*, *RPS28*, *RPS29*, and *RPS3A*.

### Dense-tiling screen execution

The screen was executed with the same protocol as the others at a maintenance coverage of approximately 12,000 K562 cells per sgRNA. After 20 days, genomic DNA was harvested and sgRNAs were amplified and sequenced with an Illumina NextSeq to a depth of 2333–3153 reads per sgRNA using the protocol described above.

### Tiling screen library design and execution

We designed an sgRNA library (referred to from now on as the tiling screen library) that would allow us to compare different CRISPR-Cas9 platforms in an unbiased fashion. To this end, we decided to focus on a limited set of genes with an already known strong growth effect, specifically *GATA1* [guides covering the genomic region chrX:48544984-48752721 (in hg19 coordinates), covering a total region of 207.737 kb, with tiling density 9308/207.737 kb = ~44 guides per kilobase], *MYB* (guides covering the genomic region chr6:135402680-135640267, covering a total region of 237.587 kb, with tiling density 9200/237.587 kb = ~38 guides per kilobase), and *ZMYND8* (guides covering the genomic region chr20:45737857-46085556, covering a total region of 347.699 kb, with tiling density of 14282/347.699 kb = ~41 guides per kilobase). These regions were determined by tiling the full annotated gene sequence and then extending the tiling for an additional 100 kb in either direction.

We filtered guides as follows. We discarded guides that had any exact or one-mismatch targets in DNase-hypersensitive sites^55^ or exons. We also filtered out sgRNAs that had any perfect matches in the genome, or >10 1-mismatch, >50 2-mismatch or >200 3-mismatch genome-wide off-targets. Matches were defined by aligning the guides to the genome using BWA ‘aln’ with the flags -N -n 4 -o 0 -k 0 -l 7^71^. The screen data presented in Fig. 3c, d and Supplementary Fig. 6 is further filtered more stringently to only display sgRNAs with no perfectly matching and no 1-mismatch off-target sites as defined by the GuideScan search algorithm.

To allow direct comparison of effect sizes of regulatory elements in the screen with those of genes, we also included guides targeting the coding regions of the 3 genes of interest (10 guides per gene). Finally, we added a set of 1000 control guides targeting safe regions as defined previously^27^.

The screen was executed with the same protocol as the others. After 14 days, genomic DNA was harvested and sgRNAs were amplified and sequenced using the protocol described above.

### Screen data analysis

The casTLE v1.0 framework^5^ was used to process screen data, including alignment of reads to an index of guide oligos, subsequent guide filtering, and estimation of effects on cell growth. For growth screens, enrichment scores were calculated by comparing samples from the final day (day 14, 21, or 23, depending on the screen) with the plasmid library.

For the CTCF motif screen, we ran makeIndices.py with parameters ‘-s 31 -e 37’ and makeCounts.py with parameters ‘-l 20’; we also grouped sgRNAs that target the same motif to measure motif-level effects and called hits using combined biological replicates with a 10% false discovery rate, using the script analyzeCombo.py. For the dense-tiling screen, we ran makeIndices.py with parameters ‘-s -34 -e 17’ and makeCounts.py with parameters ‘-l 17 -m 0 -s -’. For the tiling screen, we ran makeIndices.py with parameters ‘-s 11 -e 17’ and makeCounts.py with parameters ‘-l 19’.

### GuideScan-aggregated CFD specificity scores

We retrieved GuideScan v1.0^42^ aggregate specificity scores from the webtool. GuideScan is an off-target search algorithm that forgoes short string alignment (e.g., BWA) to find off-target locations and instead recovers locations from a pre-computed trie data structure. The webtool also reports aggregate specificity scores: these are Cutting Frequency Determination (CFD) scores (a weighted function of mismatch number, position, and nucleotide identities)^44^ for all off-target locations with 2 to 3 mismatches, that are then aggregated with the summation formula from the CRISPR MIT tool^20^ (dividing 1 by the sum of 1 plus all the CFDs), such that sgRNAs with more off-target activity approach GuideScan scores of 0. The webtool does not provide scores for sgRNAs with multiple perfect genomic matches or any off-targets that only differ by 1 mismatch, which are assumed to be too poor specificity for use in experiments, and we also excluded such sgRNAs from the analyses using GuideScan.

### Competitive growth assays

Competitive growth assays were performed with stable K562 lines expressing Cas9, CRISPRi, or CRISPRa that were lentivirally transduced with a vector (pMCB320) expressing the sgRNA and mCherry and then, after 2 to 3 days, selected with puromycin for 3 to 4 days, until the mCherry + fraction of cells was >90%. Then 40,000 of these mCherry + cells were mixed 1:1 with blank cells from the parental line (Day 0) in 1 mL of fresh RPMI media and grown in triplicate or quadruplicate in 24-well plates. The cells were maintained at a confluence less than 1e6 cells per mL. The changes in the mCherry + proportion of cells were measured on an Accuri BD C6 flow cytometer on Day 0, 4, and 7 and gating on mCherry expression in channel FL3.

### Motif mapping

Transcription factor motif recognition sequences were mapped genome-wide using FIMO^72^ (version 4.12.0 of the MEME-Suite^73^ using the CIS-BP database^74^ as a reference set of position weight matrices).

### External datasets

Data on the fitness effect of protein coding genes in K562 cells was obtained from previously published studies^5,53^. Uniformly processed ChIP-seq and DNAse-seq datasets were obtained from the ENCODE portal (https://encodeproject.org). Data on dCas9-KRAB-MeCP2 screens were retrieved from the published supplementary materials^18^. Guide-seq data were retrieved from a publication^43^ that collected off-target data from several original sources^20,21,75^.

### ChromHMM annotations

ChromHMM^76^ tracks for K562 chromatin state^41^ were retrieved from https://egg2.wustl.edu/roadmap/data/byFileType/chromhmmSegmentations/ChmmModels/coreMarks/jointModel/final/E123_15_coreMarks_mnemonics.bed.gz and visualized with the WashU Epigenome Browser^77^.

### ChIP-seq experiments

ChIP-seq experiments were carried out as described^78^ with some modifications. Briefly, 2e7 K562 cells were pelleted at 500 × *g* for 5 min at 4 °C and then resuspended in 1× PBS buffer; 37% formaldehyde solution (Sigma F8775) was added at a final concentration of 1%. Crosslinking was carried out at room temperature for 15 min, and then the reaction was quenched by adding 2.5 M Glycine solution at a final concentration of 0.25 M. Crosslinked cells then were pelleted 500 × *g* for 5 min at 4 °C, washed with cold 1× PBS buffer, and stored at −80 °C.

CTCF ChIP was performed using a polyclonal anti-CTCF antibody (Millipore, 07–729). For each reaction, 100 µL of Protein A Dynabeads (Thermo Fisher 10001D) were washed 3 times with a 5 mg/mL BSA (Sigma A9418) solution. Beads were then resuspended in 1 mL BSA solution and 4 µL of CTCF antibody were added. Coupling of antibodies to beads was carried out overnight on a rotator at 4 °C. Beads were again washed 3 times with BSA solution, resuspended in 100 µL of BSA solution, mixed with 900 µL sonicated chromatin and incubated overnight on a rotator at 4 °C. Chromatin was sonicated using a tip sonicator (Misonix) after cells were lysed with Farnham Lysis Buffer (5 mM HEPES pH 8.0, 85 mM KCl, 0.5% IGEPAL, Roche Protease Inhibitor Cocktail), and nuclei were resuspended in RIPA buffer (1× PBS, 1% IGEPAL, 0.5% Sodium Deoxycholate, 0.1% SDS, Roche Protease Inhibitor Cocktail). The sonicated material was centrifuged at 14,000 rpm at 4 °C for 15 min to remove cellular debris, and a portion of the supernatant was saved as input. After incubation with chromatin, beads were washed 5 times with LiCl buffer (10 mM Tris-HCl pH 7.5, 500 mM LiCl, 1% NP-40/IGEPAL, 0.5% Sodium Deoxycholate) by incubating for 10 min at 4 °C on a rotator and then rinsed once with 1× TE buffer. Beads were then resuspended in 200 µL IP Elution Buffer (1% SDS, 0.1 M NaHCO_3_) and incubated at 65 °C in a Thermomixer (Eppendorf) with interval mixing to dissociate antibodies from chromatin. Beads were separated from chromatin by centrifugation, Proteinase K was added to the supernatant and crosslinks were reversed at 65 °C for ~16 h. Input samples (100 µL) were mixed with an equal volume of IP Elution Buffer, Proteinase K was added and cross-links were reversed together with the ChIP samples. DNA was purified by phenol-chloroform-isoamyl extraction followed by MinElute column (Qiagen) clean up. DNA concentration was measured using QuBIT, and libraries were generated using the NEBNext Ultra II DNA Library Prep Kit for Illumina (NEB, E7645S). Libraries were sequenced on a NextSeq (Illumina) in a 2 × 75 bp format.

### ChIP-seq data processing

Demultipexed fastq files were initially mapped to the hg19 assembly of the human genome (female version) as 1 × 36mers using Bowtie v1.0.1^79^ with the following settings: ‘-v 2 -k 2 -m 1 --best --strata’, for quality assessment purposes (see AQUAS: https://github.com/kundajelab/chipseq_pipeline) (Supplementary Table 2). For subsequent analyses of CTCF occupancy, reads were mapped against the female version of the hg19 assembly of the human genome using the ‘bwa mem’ algorithm in the BWA aligner with default settings and filtering non-unique and low-quality alignments using samtools^71^ with the ‘-F 180 -q 30’ options. A consensus set of peaks was derived from the three safe sgRNA CTCF ChIP-seq datasets as described in the AQUAS pipeline. FRiP values^80^ were calculated for each dataset using this set of peak calls. Our peak set overlapped by 82–89% with different available ENCODE K562 CTCF ChIP-seq peak sets, while the ENCODE samples overlapped with one another by 73–94%. Read coverage tracks were generated using custom-written Python scripts. For the purpose of comparison between datasets and normalizing for differences in ChIP strength between individual experiments, tracks were rescaled as follows:

1 $${C_{{\mathrm{chr}},i}}^ \ast \left( D \right) = C_{{\mathrm{chr}},i}\left( D \right) \ast \frac{{{\mathrm{max}}_D\left( {{\mathrm{FRIP}}} \right)}}{{{\mathrm{FRIP}}_D}}$$

Where $C_{{\mathrm{chr}},i}(D)$ is the normalized coverage (in RPM, or Read Per Million mapped reads units) of position *i* on a given chromosome chr in dataset *D*, and ${C_{{\mathrm{chr}},i}}^ \ast (D)$ is the rescaled coverage.

### RNA-seq experiment

2e7 K562 cells per replicate were pelleted at 500*×g* for 5 min at 4 °C and then resuspended in 1× PBS buffer. Two replicates were performed for each sgRNA. RNA extraction was performed as follows: 500 µL of TRIzol was added to each sample, mixed by inverting the tube, and then 5 min later 100 µL of chloroform was added. Samples were spun at 12,000 × *g* for 15 min at 4 °C. The aqueous layer was transferred to an RNase-free tube and mixed with 300 µL of 70% ethanol and vortexed. Contents were then transferred to Direct-zol Miniprep columns (Zymo) and the protocol was followed according to the manufacturer’s instructions, including the on-column DNaseI treatment. RNA was eluted in 15 µL of RNase-free water and stored at −80 °C and a separate 2 µL aliquot was set aside for testing RNA concentration and quality via Nanodrop. RNA-seq libraries were prepared from 700 ng of total RNA with the TruSeq RNA Library Prep kit v2 (Illumina) low sample protocol, which uses oligo-dT beads to enrich for A-tailed mRNAs. Library concentration and length was determined with a 2200 Tapestation System (Agilent) and Qubit (Thermo Fisher Scientific). Libraries were pooled and sequenced on a Nextseq (Illumina).

### RNA-seq data processing and analysis

Paired-end 2 × 50 bp RNA-seq reads were mapped using version 2.5.3a of the STAR aligner^81^ against the hg19 version of the human genome with haplotypes removed but retaining random chromosomes, with version 19 of the GENCODE annotation^70^ as a reference. Gene expression quantification was then carried out on the STAR alignments transformed into transcriptome space using version 1.3.0 of RSEM^82^. Differential expression analysis was performed using DESeq2^83^ with the RSEM estimated read counts per gene as an input. Mapping and QC statistics are provided in Supplementary Table 3.

### ATAC-seq experiments

ATAC-seq experiments were carried out following the Omni-ATAC-seq protocol^84^, using two replicates per sgRNA. Briefly, cells were pretreated with 200 U/ml DNase (Worthington) for 30 min at 37 °C, then washed, resuspended in cold PBS, and counted. 50,000 cells were resuspended in 1 ml of cold ATAC-seq resuspension buffer (RSB; 10 mM Tris-HCl pH 7.4, 10 mM NaCl, and 3 mM MgCl_2_ in water). Cells were centrifuged at 500×*g*. for 5 min in a pre-chilled (4 °C) fixed-angle centrifuge. Cell pellets were then resuspended in 50 μl of ATAC-seq RSB containing 0.1% NP40, 0.1% Tween-20, and 0.01% digitonin and incubated on ice for 3 min. After lysis, 1 ml of ATAC-seq RSB containing 0.1% Tween-20 was added, and the tubes were inverted to mix. Nuclei were then centrifuged for 10 min at 500 × *g*. At 4 °C. Supernatant was removed and nuclei were resuspended in 50 μl of transposition mix (25 μl 2× TD buffer, 2.5 μl transposase (100 nM final), 16.5 μl PBS, 0.5 μl 1% digitonin, 0.5 μl 10% Tween-20, and 5 μl water). Transposition reactions were incubated at 37 °C for 30 min in a thermomixer with shaking at 1000 r.p.m. Reactions were cleaned up with Zymo DNA Clean and Concentrator 5 columns. The ATAC-seq library was then subjected to PCR amplification  with NEBNext (NEB, M0541) for 10-25x cycles (with the minimal sufficient cycle number determined by qPCR as described^85^), purified with a MinElute column (QIAGEN, 28004), and sequenced on an Illumina NextSeq.

### ATAC-seq analysis

Paired-end 2 × 36 bp reads were first mapped to the mitochondrial genome to assess the fraction of mitochondrial reads in each sample. All other reads were then mapped to the hg19 genome assembly using BWA as described above. Statistics are summarized in Supplementary Table 4.

### ICE analysis of indels

Cells were harvested and total genomic DNA was isolated using QuickExtract DNA Extraction Solution (VWR, Radnor, PA, cat# QE09050). PCR was prepared using 5X GoTaq Green Reaction Buffer and GoTaq DNA Polymerase (Promega, Madison, WI, cat# M3005), 10 mM dNTPs, and primers designed approximately 250–350 basepairs upstream and 450–600 basepairs downstream of the predicted cut site. PCR reactions were run on a C1000 Touch Thermo Cycler (Bio-Rad). PCR products were then purified over an Econospin DNA column (Epoch, Missouri City, TX, cat# 1910-250) using Buffers PB and PE (Qiagen, Hilden, Germany, cat# 19066 and cat# 19065). Sanger sequencing ab1 data were obtained from Quintara Biosciences and editing efficiency of knockout cell lines were analyzed using Synthego’s online ICE Analysis Tool (https://ice.synthego.com)^86^.

### RT-qPCR experiments

RNA from 100,000 K562 cells was extracted with RNA QuickExtract (Lucigen QER090150). RNA was treated with DNaseI from the same kit, reverse transcribed with AMV RT (Sigma 10109118001), and then cDNA were quantified in multiplex TaqMan qPCR reactions using commercially available probe sets (Thermo Fisher 4453320) and TaqMan FastAdvanced Master mix (Thermo Fisher 4444556). Three to four technical qPCR replicates were used for each biological replicate.

### Flow cytometry for GATA1 protein levels

We devised a flow cytometry assay wherein we co-culture cells expressing the sgRNA and mCherry from a lentivirus with non-transduced cells and stain for GATA1 protein. Intracellular staining of GATA1 protein levels was performed using a previously published method^87^. Specifically, cells were fixed with Fix Buffer I (BD Biosciences) for 15 min at 37 °C. Cells were washed with 10% FBS in PBS once and then permeabilized on ice for 30 min using Perm Buffer III (BD Biosciences). Cells were washed twice and then stained with anti-GATA1 primary (1:1000, rabbit, Cell Signaling Technologies cat no. 3535 S) for 1 h at 4 °C. After two more washes, cells were incubated with Goat anti-rabbit antibody conjugated to Alexa Fluor 647 (1:1000, ThermoFisher cat no. A-21244) for 1 h at 4 °C. After a final round of washing, flow cytometry was performed using a FACScan flow cytometer (BD Biosciences). We analyzed the data with CytoFlow by gating the cells on mCherry expression and then plot the GATA1 protein level in mCherry + and non-transduced cells. This approach controls for variability in staining efficiency as the two cell groups are mixed within the same sample.

### Western blot for GATA1 protein levels

Cells transduced with a lentiviral vector containing an sgRNA and puromycin-T2A-mCherry were selected with puromycin (1 μg/mL) until mCherry was >85%. 1 million cells were lysed in lysis buffer (1% Triton X-100, 150 mM NaCl, 50 mM Tris pH 7.5, 1 mM EDTA, Protease inhibitor cocktail). Protein amounts were quantified using the DC Protein Assay kit (Bio-Rad). Equal amounts were loaded onto a gel and transferred to a nitrocellulose membrane. Membrane was probed using GATA1 antibody (1:1000, rabbit, Cell Signaling Technologies cat no. 3535 S) and GAPDH antibody (1:2000, mouse, ThermoFisher cat no. AM4300) as primary antibodies. Donkey anti-rabbit IRDye 680 LT and goat anti-mouse IRDye 800CW (1:20,000 dilution, LI-COR Biosciences, cat nos. 926–68023 and 926–32210, respectively) were used as secondary antibodies. Blots were imaged on a LiCor Odyssey CLx. Uncropped images are provided in the Source Data file.

### Reporting summary

Further information on research design is available in the Nature Research Reporting Summary linked to this article.

## Supplementary information

Supplementary Information

Reporting Summary

Description of Additional Supplementary Files

Supplementary Data 1

Source Data

## Data Availability

The following datasets are accessible in the online GEO repository with accession GSE131349: CRISPR-Cas9 screen data (tiling screens, dense-tiling screen, CTCF motif screens), CTCF ChIP-seq, ATAC-seq, RNA-seq [GSE131349]. Source data for the figures provided are available in the Source Data file. All other relevant data are available from the authors upon reasonable request.

## Code availability

Python scripts are available on GitHub for library design and guide scoring. (1) Library design: extractGuidesFromGuideScan.py takes a list of regions and returns a desired number of guides within each of those regions, filtering for either the number of off-targets or the GuideScan specificity scores. (2) Screen analysis: GuidesPerRegionFromWholeGenomeGuideScan.py takes a list of sgRNA sequences and returns their GuideScan specificity scores (https://github.com/georgimarinov/non_coding_CRISPR_screen_design). The underlying data, GuideScan scores for all sgRNAs in the human hg38 genome and mouse mm10 genome, were downloaded from the Guidescan webtool (www.guidescan.com), and are also provided to enable direct batch processing.
