# Supplementary material for: Mitigation of off-target toxicity in CRISPR-Cas9 screens for essential non-coding elements
Source: Nat Commun. 2019 Sep 6;10:4063. doi: 10.1038/s41467-019-11955-7 (PMC6731277; doi:10.1038/s41467-019-11955-7)
Supplement: Supplementary file 1 — Supplementary Information [file 41467_2019_11955_MOESM1_ESM.pdf]

Supplementary Information

Mitigation of off-target toxicity in CRISPR-Cas9 screens for essential non-coding elements

Josh Tycko*, Michael Wainberg*, Georgi K. Marinov*, Oana Ursu, Gaelen T. Hess, Braeden K. Ego, Aradhana, Amy Li, Alisa Truong, Alexandro E. Trevino, Kaitlyn Spees, David Yao, Irene M. Kaplow, Peyton G. Greenside, David W. Morgens, Douglas H. Phanstiel, Michael P. Snyder, Lacramioara Bintu, William J. Greenleaf#, Anshul Kundaje#, Michael C. Bassik#

* These authors contributed equally to this work

\# Correspondence should be addressed to M.C.B. (bassik@stanford.edu), A.K. (akundaje@stanford.edu), or W.J.G. (wjg@stanford.edu).

SUPPLEMENTARY FIGURE 1

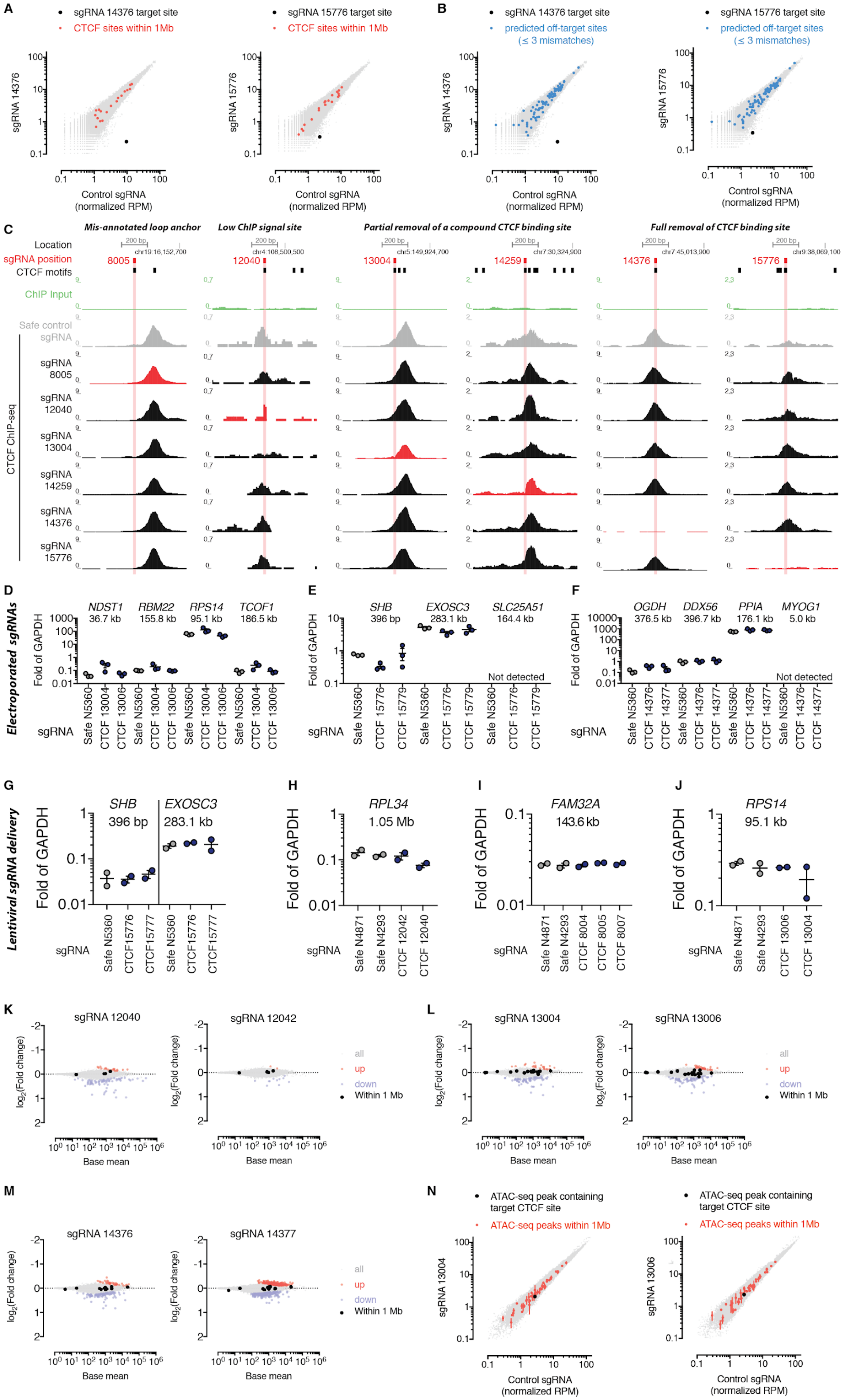

Supplementary Figure 1. Follow-up studies of individual sgRNAs targeting CTCF motifs.

A. CTCF ChIP-seq was performed on the K562 cells stably expressing a CTCF-targeting sgRNA. No other CTCF peaks within 1 Mb of the on-target location were significantly affected.
B. No other CTCF peaks that overlap a predicted off-target site with ≤3 mismatches were affected. List of off-target sites was provided by the Cas OFFinder webtool [1].
C. CTCF ChIP-seq was performed on the K562 cells stably expressing a CTCF-targeting sgRNA. Each column presents a particular CTCF ChIP peak and the red track highlights the sgRNA that has an on-target match in that column. While some sgRNAs completely ablate CTCF binding, others only remove part of a compound CTCF ChIP peak. sgRNA 8005 targets a motif that was not in fact underlying the nearest ChIP-seq peak, likely due to problems with motif annotation or differences between K562 cell lines, yet this guide still confers a validated growth phenotype.
D. No significant changes in the expression of nearby essential genes were detected for any of the CTCF-targeting sgRNA that were individually tested. sgRNA-mCherry plasmids were delivered by electroporation, 36 hours later the cells were confirmed to be > 70% mCherry+ by flow cytometry and RNA was extracted for qPCR. *NDST1* is a non-essential gene and the CTCF motif falls within one of its introns. *RBM22, RPS14,* and *TCOF1* are the nearest essential genes. The distances shown below the gene names are between the CTCF motif and the TSS of the gene.
E. *SHB* is a non-essential gene and the CTCF motif falls within its 5′ UTR; *EXOSC3* and *SLC25A51* are the nearest essential genes.
F. *MYOG1* is a non-essential gene and the CTCF motif falls within its intron. *OGDH*, *DDX56, and PPIA* are the nearest essential genes. Genes are determined to be essential if they were called as hits with a 10% FDR in previous Cas9 [2], or CRISPRi/a gene screens [3].
G. Individual sgRNAs were delivered by lentivirus, 2 days later cells were selected for sgRNA delivery with puromycin, and 5 days after delivery RNA was extracted for qPCR. Both sgRNAs labeled “CTCF” (i.e. sgRNAs 15776 and 15777) target the same CTCF motif. Same target motif as in **D**.
H. *RPL34* is the nearest essential gene.
I. *FAM32A* is the nearest essential gene.
J. *RPS14* is the nearest essential gene..
K. The lenti-transduced cells were subjected to RNA-seq and the mRNA expression fold-changes compared to safe-targeting sgRNAs is shown. The two sgRNAs target the same CTCF motif. None of the black dots (genes within 1 Mb of the motif) are significantly differentially expressed.
L. As in **J** for another target CTCF motif.
M. As in **J** for another target CTCF motif.
N. No changes in ATAC-seq peaks in the cells stably expressing CTCF-targeting sgRNAs 13004 or 13006.

SUPPLEMENTARY FIGURE 2

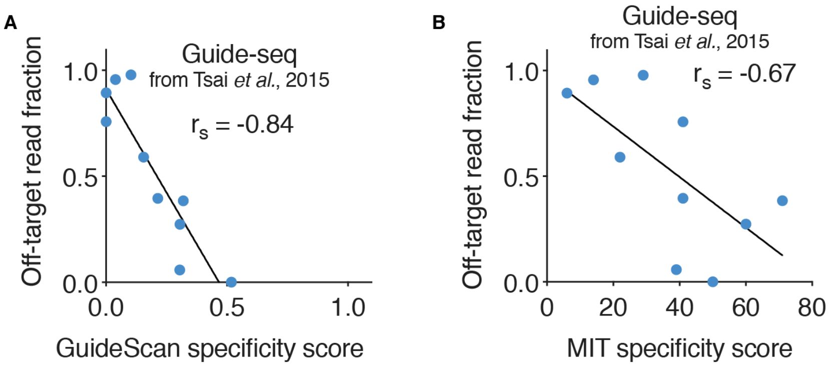

Supplementary Figure 2. Validation of GuideScan aggregated CFD specificity scores with off-target activity assays.

A. We retrieved GuideScan specificity scores for sgRNAs that were tested for off-target cleavage with the unbiased, genome-wide assay Guide-seq [4]. The scores correlate with the off-target read fraction, defined as the fraction of total Guide-seq reads that align to off-target sites. Some sgRNAs did not have GuideScan scores because they had multiple perfect genomic matches or off-targets with only 1 mismatch; these sgRNAs were given a score of 0 for this analysis.

B. MIT specificity scores [5], as reported in a meta-analysis of these off-target studies [6] were compared with Guide-seq as in (A).

SUPPLEMENTARY FIGURE 3

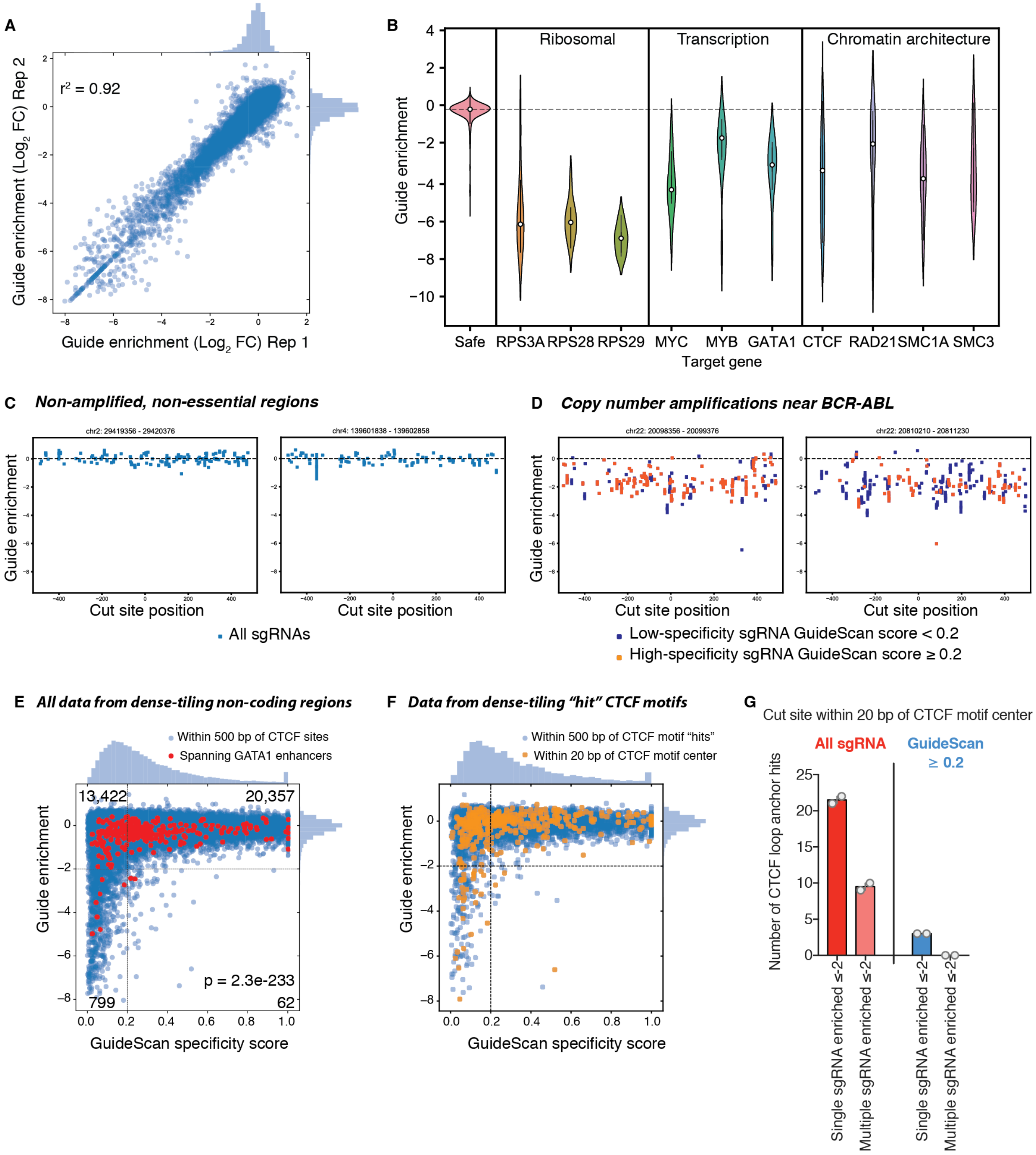

Supplementary Figure 3. Dense-tiling screen confirms confounding effect of off-target activity.

A. Reproducibility of biological replicates from a growth screen using the dense-tiling library.

B. Positive controls demonstrate successful detection of essential genes. The targeted genes are essential [7], meaning that targeting them should decrease cell growth. Each gene was targeted with 10 sgRNAs in its coding regions; the distribution of sgRNAs is shown, and the functional annotation of each gene is labeled. Safe arerefers to safe-targeting negative control sgRNA.

C. Examples of two non-amplified regions without any essential elements or any sgRNA confounded by off-target activity.

D. Examples of two copy number amplified regions near *BCR-ABL* showing a distinct uniform depletion that is unrelated to the specificity of the sgRNAs.

E. Low-specificity sgRNAs, in both the CTCF-anchor and GATA1-enhancer regions, are significantly enriched to have growth effects (p-value from Fisher's exact test).

F. Shown is the subset of the dense-tiling screen from 1 kb windows around motifs that previously had evidence of strong essentiality in the CTCF motif-targeting screen.

G. There were no CTCF motifs with concordant evidence of fitness effects from multiple high-specificity sgRNAs, despite targeting 37 CTCF motifs with multiple high-specificity sgRNA and these CTCF sites previously being called as “hits” in the CTCF motif-targeting sgRNA screen. Grey circles are screen biological replicates and the bar marks the mean value.

SUPPLEMENTARY FIGURE 4

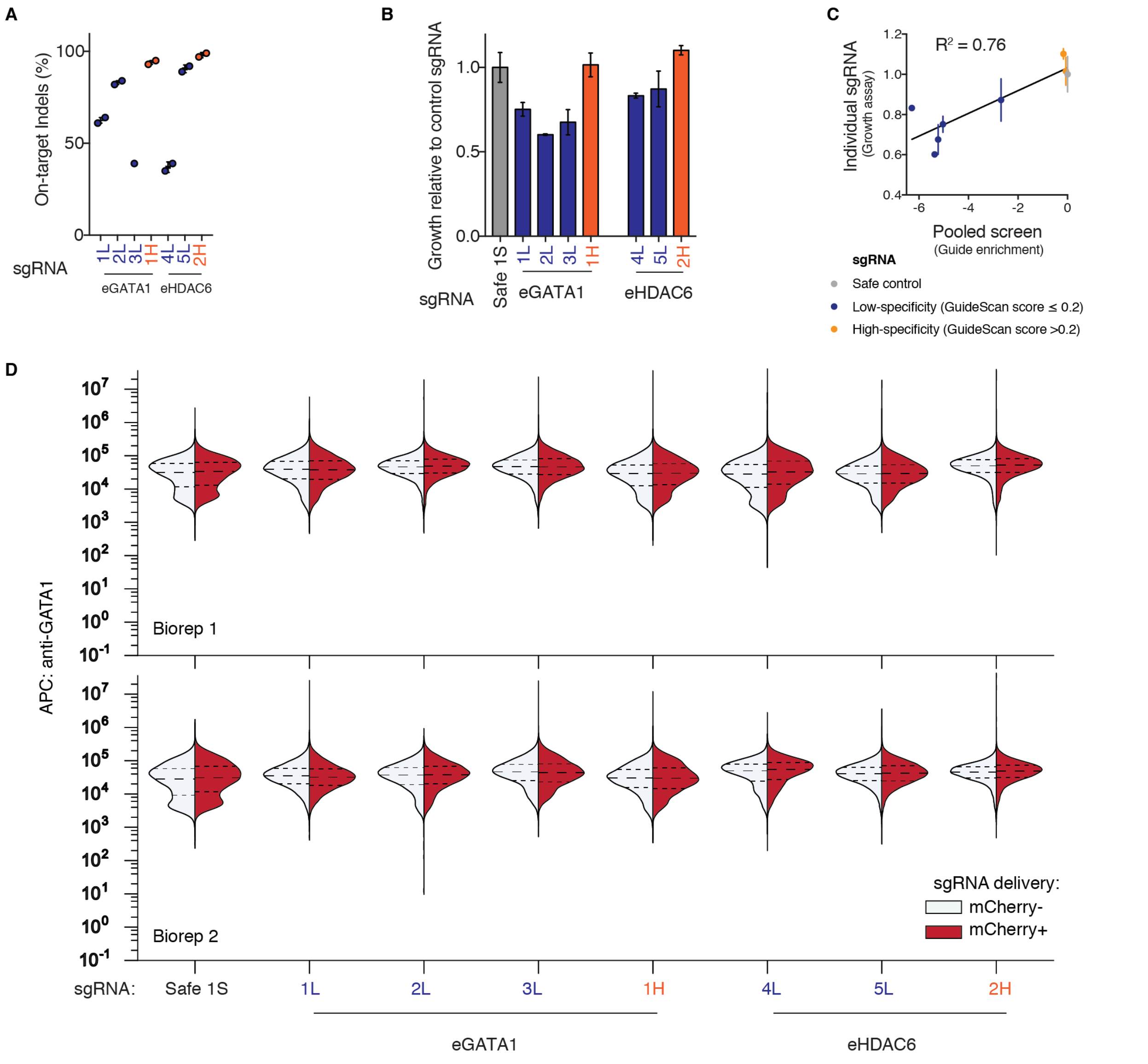

Supplementary Figure 4. Validation experiments for dense-tiling screen of enhancers of *GATA1*.

A. Individual sgRNAs generated on-target indels in K562 after lentiviral delivery and puromycin selection, as quantified by ICE analysis [8,9]. Dark blue dots correspond to sgRNAs that reduce fitness and have low GuideScan scores, and orange dots correspond to sgRNAs that do not reduce fitness and have high GuideScan scores.

B. Competitive growth assay validated expected growth effects in these individual cell lines.

C. Individually measured growth effects correlate with the pooled screen measurements.

D. Additional flow cytometry for GATA1 protein levels confirmed there was no change in expression of GATA1 in these cell lines. Cells transduced with the sgRNA-mCherry lentiviral vector were co-cultured with non-transduced parental cells and then stained and analyzed by FACS together in order to control for variation in staining efficiency between samples. In all samples, the distribution of GATA1 levels is not significantly different between the mCherry+ and blank cells. Dashed lines within the histograms mark the quartiles. sgRNA labeled as in **Figure 2**.

SUPPLEMENTARY FIGURE 5

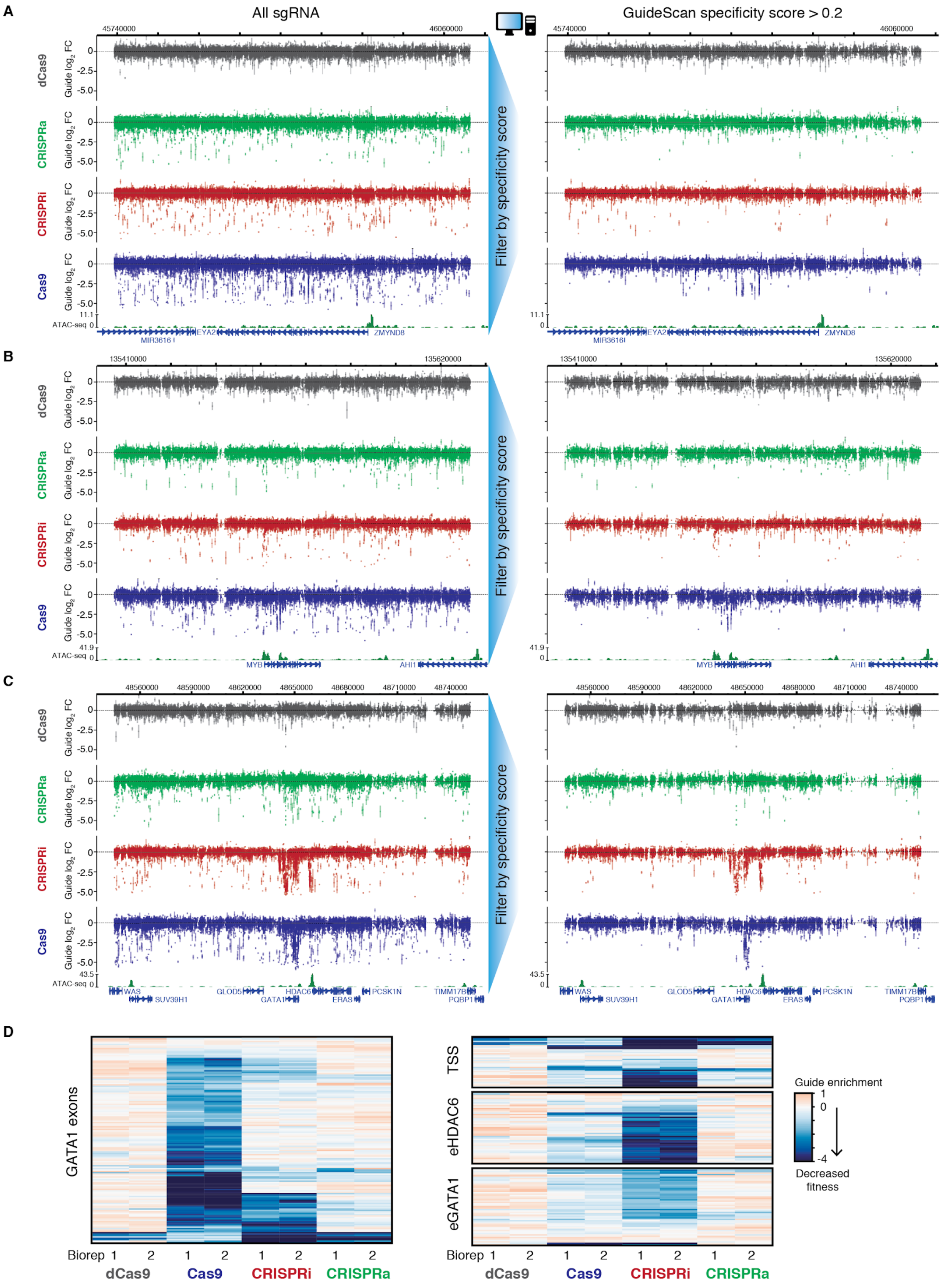

Supplementary Figure 5. Tiling screens of three regions around essential genes with four CRISPR-Cas9 perturbations.

A. Four parallel screens were conducted tiling the loci of essential growth genes *GATA1, MYB,* and *ZMYND8* using the four platforms Cas9, CRISPRa, CRISPRi and dCas9. Shown is the full tiled region around *ZMYND8* with and without filtering for high-specificity sgRNAs with the GuideScan score.
B. Full tiled region around *MYB*.
C. Full tiled region around *GATA1*.
D. Clustering of sgRNAs from the *GATA1* tiling screen that target regions with expected on-target effects (exons, TSS, and enhancers).

SUPPLEMENTARY FIGURE 6

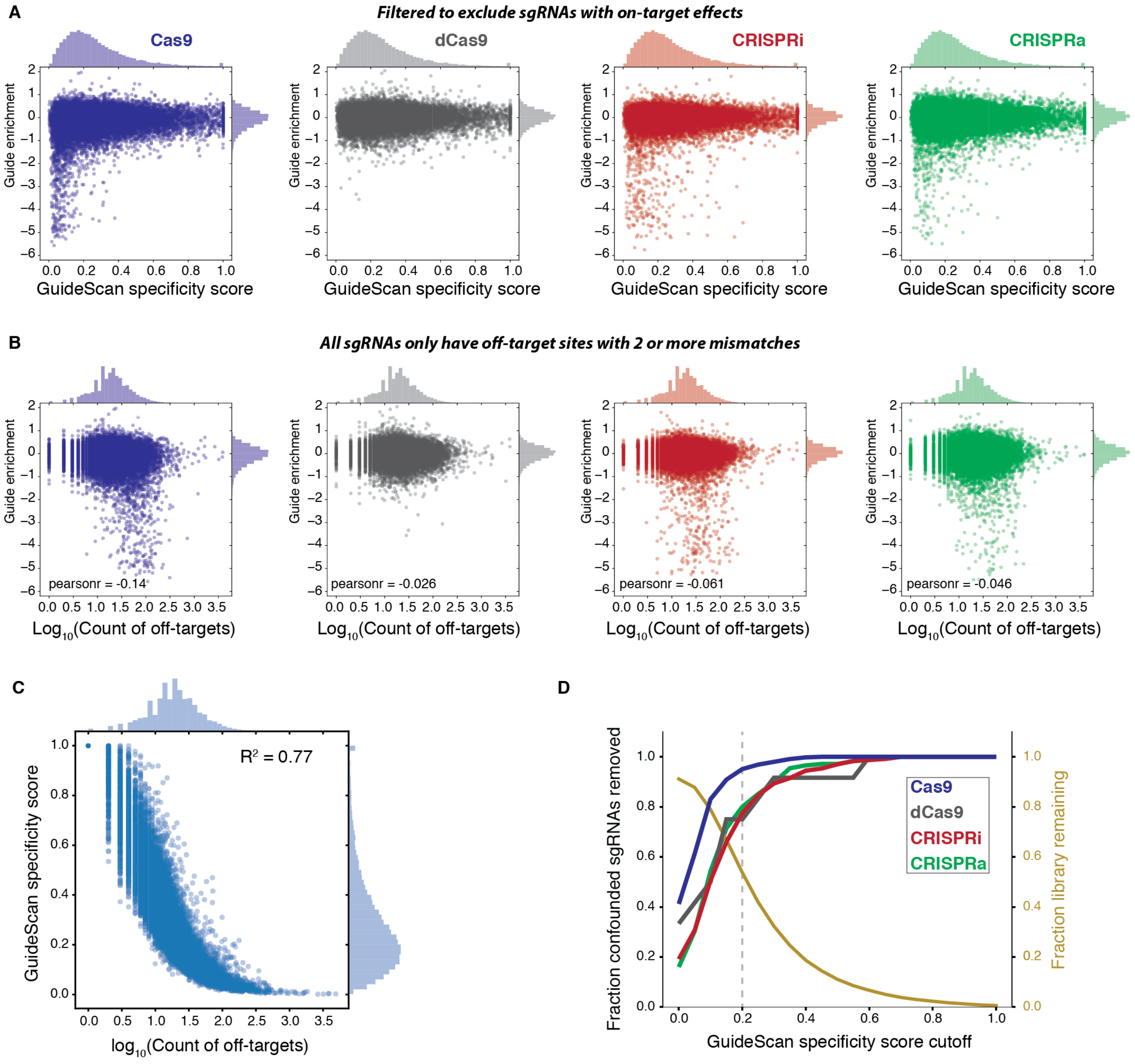

Supplementary Figure 6. Comparison of fitness effects and specificity scores with the number of off-target binding locations.

A. Comparison of GuideScan scores with fitness effects in the tiling screen, filtered to exclude sgRNAs that are likely to have on-target growth effects by removing sgRNAs 1000 bp upstream to 1000 bp downstream of *ZMYND8* or *MYB* coding sequences, and 1000 bp upstream of eGATA1 to 1000 bp downstream of eHDAC6. For the similar plot that includes those sgRNAs, see **Figure 3C**. sgRNAs with multiple perfect matches to the genome or off-target locations with only 1 mismatch were excluded.

B. For the same set of sgRNAs in **A**, we compared the guide enrichment from the tiling screen with the number of off-target binding locations that have 2-3 mismatches. The off-target search was done with GuideScan.

C. For comparison, the relationship between the GuideScan specificity score and the number of off-target locations for the same sgRNAs in the tiling screen library.

D. Tradeoff between removing confounded sgRNAs and retaining sgRNA library density. Confounded sgRNAs were defined as the set from **A** with fitness effects ≤-2. sgRNAs with any perfectly-matched or 1-mismatch off-target sites are considered to have GuideScan scores <0 for this analysis.

SUPPLEMENTARY FIGURE 7

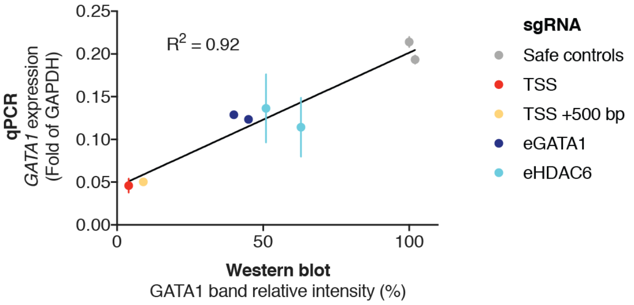

Supplementary Figure 7. Validation of CRISPRi repression of essential enhancers with high-specificity sgRNAs.

After delivery of individual sgRNA by lentivirus, followed by puromycin selection, we performed qPCR for GATA1 mRNA levels and a Western blot for GATA1 protein levels (shown in **Figure 3**). The knockdown measurements are correlated.

SUPPLEMENTARY FIGURE 8

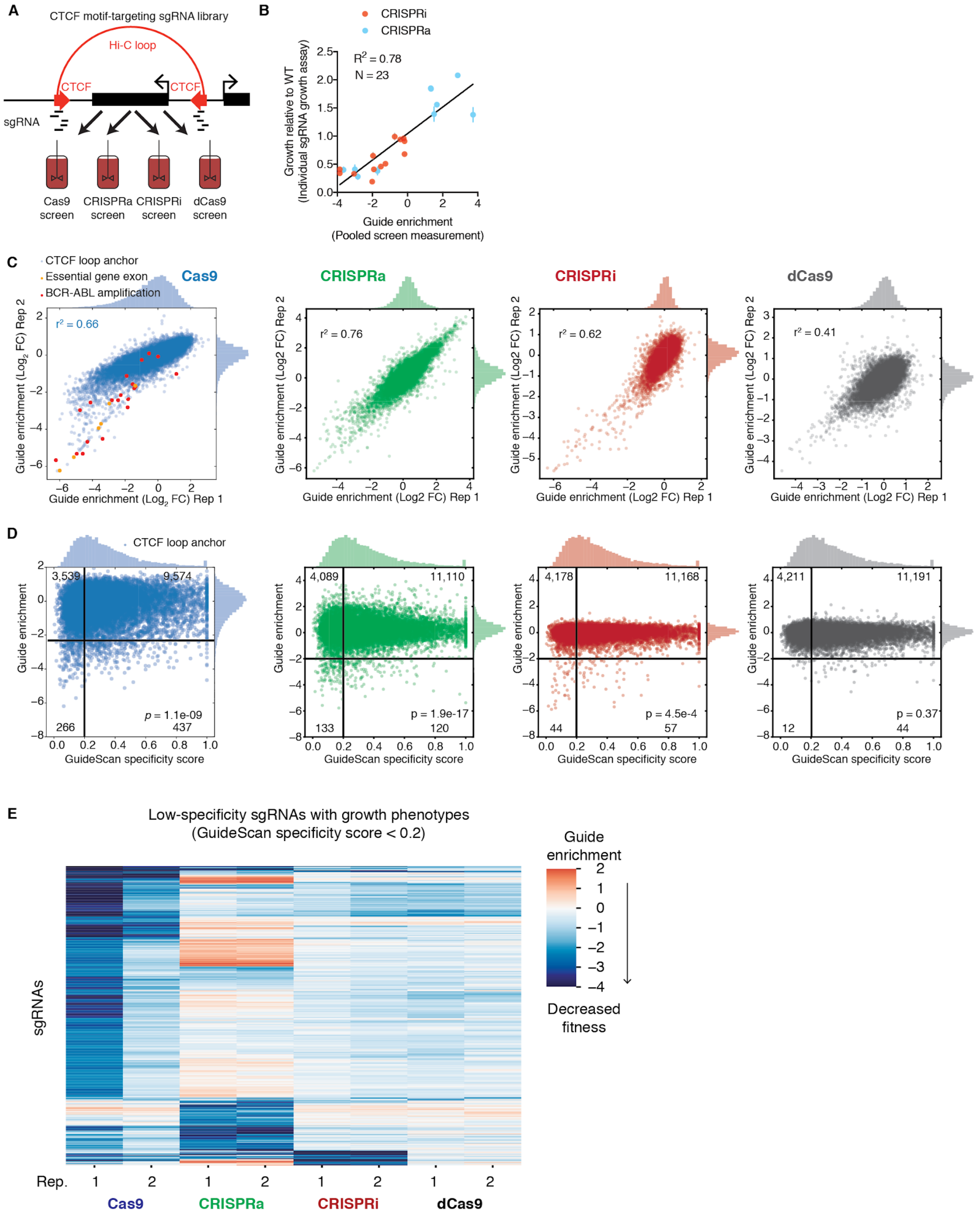

Supplementary Figure 8. Parallel screens of CTCF loop anchors with Cas9, CRISPRi/a, and dCas9.

A. The CTCF motif-targeting sgRNA library was used in parallel screens to compare the CRISPR-Cas9 platforms. All screens shown here were maintained at 3000x coverage (cells per sgRNA), whereas the Cas9 screens shown in **Figure 1** were maintained at 11,000x coverage.

B. Growth effects measured in this screen were validated with individual competitive growth assays. Validation of Cas9 effects shown in **Figure 1**. Error bars are standard deviation of three technical replicates.

C. Reproducibility between biological replicates. For CRISPRi/a, sgRNAs ≤1000 bp from the TSS of an essential gene identified in a previous CRISPRi/a gene screen were excluded to avoid on-target artifacts.

D. Low-specificity guides are significantly enriched among CTCF motif-targeting guides with fitness effects when using CRISPRi/a. P-value from Fisher's exact test, using a 2x2 table of the numbers of guides in each quadrant based on the thresholds drawn in black lines. Numbers in corners correspond to the number of CTCF site-targeting guides in the quadrant. sgRNAs with > 1 perfect matches to the genome or > 0 off-target locations with only 1 mismatch were excluded from this analysis, as before. Notably, the Cas9 screen shown here was maintained at lower coverage and thus resulted in noisier data than the replicates shown in **Figure 1**. It showed a significant, but less pronounced, enrichment for low-specificity guides among the guides with fitness effects (Fisher's exact test) than in the higher quality screen data shown in **Figure 1**, showing that experimental noise can disguise the confounding effect of off-target activity.

E. Clustering of low-specificity sgRNAs reveals that each perturbation has off-target activity that reduces cell fitness with a unique subset of the low-specificity sgRNAs. Shown are the subset of low-specificity sgRNAs that have a guide enrichment ≤-2 in at least one replicate.

SUPPLEMENTARY FIGURE 9

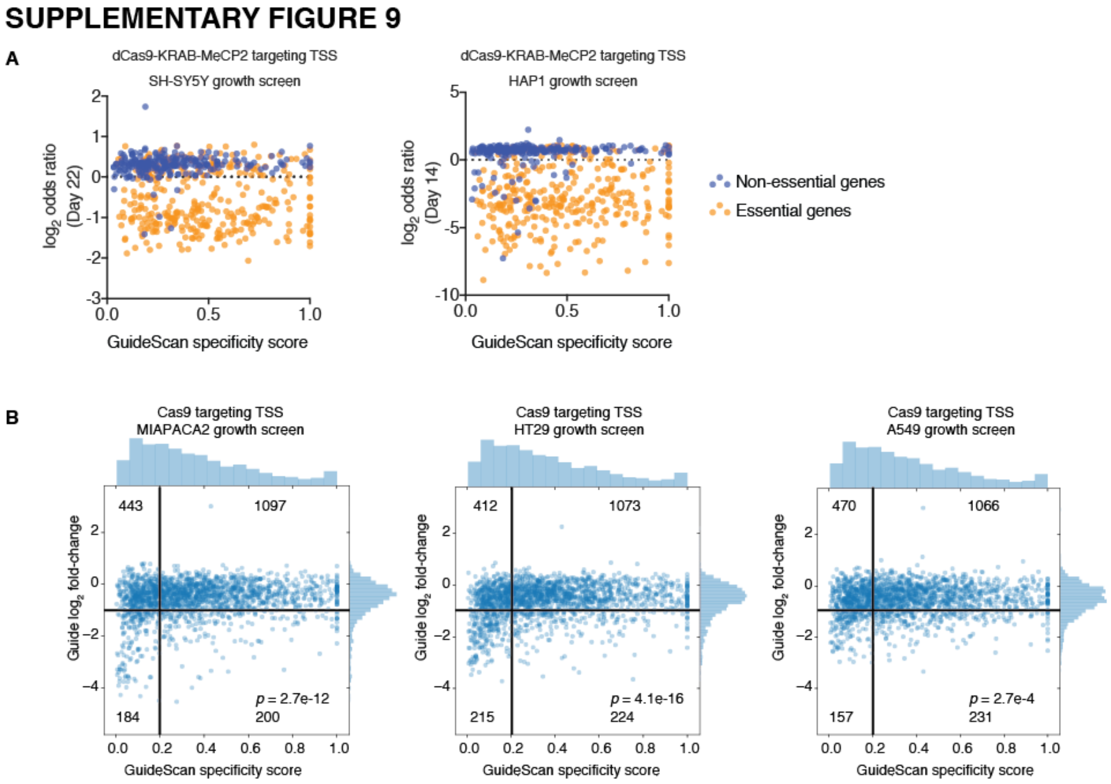

Supplementary Figure 9. sgRNAs with low GuideScan scores are enriched in growth screens in other cell types.

A. We retrieved data from a published growth screen where sgRNAs were targeted to the TSS of known essential and non-essential genes [10], in different cell types. The marked depletion of sgRNAs targeting non-essential genes was unexpected and the authors discussed the need for further investigations to clarify the source of these effects. Here, we found that these sgRNAs have low specificity scores, implicating off-target activity. However, the enrichment was not significant, possibly due to the small number of sgRNAs in the dataset.

B. We retrieved data from a published growth screen where sgRNAs targeted the TSS of genes with Cas9 [11]. We excluded sgRNAs with any off-target sites with only 0 or 1 mismatch as determined by the GuideScan search tool. There is a significant enrichment for fitness effects with low-specificity sgRNAs (Fisher's exact test).

SUPPLEMENTARY FIGURE 10

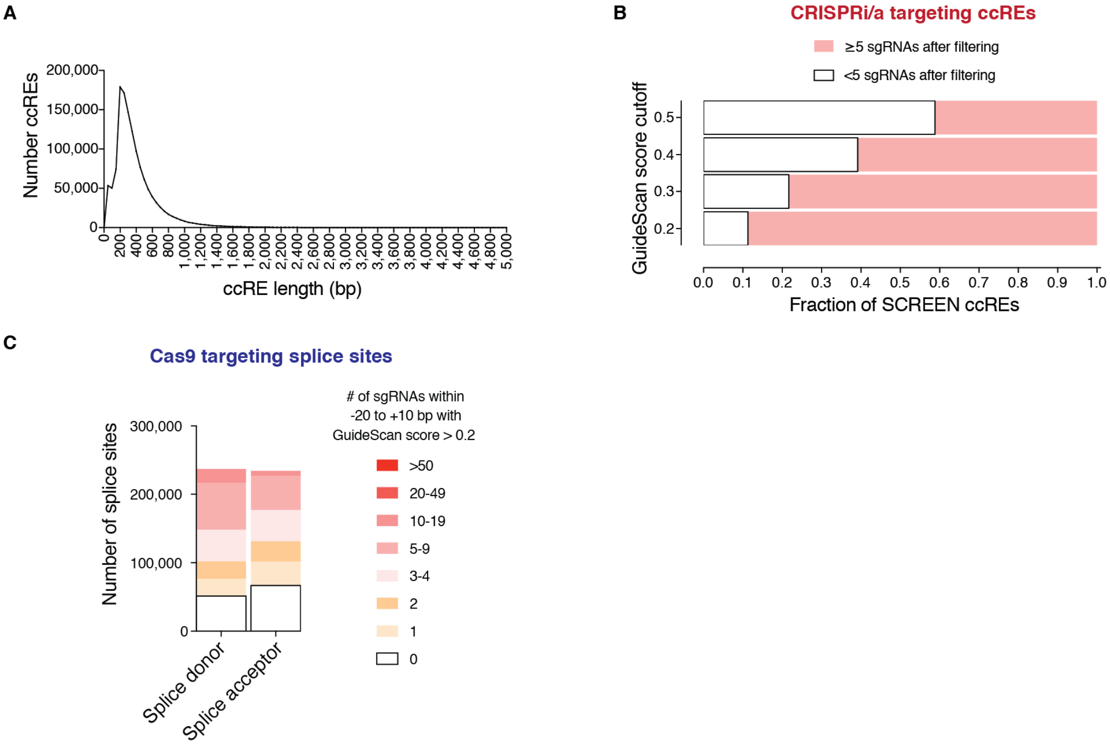

Supplementary Figure 10. Filtered library designs for regulatory elements and splice sites.

A. ccREs were retrieved from the ENCODE SCREEN database and their distribution of lengths is shown.

B. Various GuideScan score filtering cutoffs were applied to the sets of sgRNAs overlapping the ccREs. 89% of ccREs can be targeted with ≥5 sgRNAs with GuideScan scores > 0.2, enabling CRISPRi/a screens of ccREs with high-specificity libraries.

C. Fraction of splice sites that can be targeted with sgRNAs within a window (-20 to +10 bp), after filtering out low-specificity sgRNAs.

Supplementary Data

Supplementary Data 1: sgRNA libraries used in this study

The sgRNA sequences and the sgRNA scores from GuideScan are provided in a separate Excel file.

Supplementary Tables

Supplementary Table 1: sgRNA sequences from validation experiments, primers, and plasmids

sgRNA N4871 (Safe - Cas9, CRISPRi, CRISPRa)	GGAAAATGATGGTCTGCAAC
sgRNA N5360 (Safe - Cas9, CRISPRi, CRISPRa)	GCACATTTGGATTTCATGTC
sgRNA N4293 (Safe - Cas9, CRISPRi, CRISPRa)	GAGGAGAGCCAATGATCTCT
sgRNA N5284 (Safe - Cas9, CRISPRa)	GTGTCCTTGTTTAGAAAGCA
sgRNA 13004 (CTCF - Cas9)	GAGAGGGGGCCTCCAGAGGG
sgRNA 13006 (CTCF - Cas9)	GGAGCAGAGGGGGCCTCCAG
sgRNA 15776 (CTCF - Cas9, CRISPRi)	GAGCTGCCGGCAGGAGGCGG
sgRNA 15777 (CTCF - Cas9, CRISPRi)	GGAGAGCTGCCGGCAGGAGG
sgRNA 15779 (CTCF - Cas9)	GGAGCCAGAGAGCTGCCGGC
sgRNA 14376 (CTCF - Cas9)	GTTCCCAGGGGCTCCCACCA
sgRNA 14377 (CTCF - Cas9)	GTCCCCCTGGTGGGAGCCCC
sgRNA 12040 (CTCF - Cas9)	GCCCACCAGGGAGCAGCATG
sgRNA 12042 (CTCF - Cas9)	GGCCCCATGCTGCTCCCTGG
sgRNA 8004 (CTCF - Cas9, CRISPRi)	GCTAGCCAAAGGACCAGGAG
sgRNA 8005 (CTCF - Cas9, CRISPRi)	GTAGCCAAAGGACCAGGAGA
sgRNA 8007 (CTCF - Cas9, CRISPRi)	GGCCAAAGGACCAGGAGAGG
sgRNA 14259 (CTCF - Cas9)	GCGTGGGAGCCGGAGGATGG

sgRNA 14261 (CTCF - Cas9)	GGGGAGCCGGAGGATGGCGG
sgRNA 15923 (CTCF - CRISPRi)	GTGGTTGAGGGACCAGGAGG
sgRNA 15926 (CTCF - CRISPRi)	GTTGTGGTTGAGGGACCAGG
sgRNA 15699 (CTCF - CRISPRi)	GTATTCTAGCACTTGCCCAC
sgRNA 15703 (CTCF - CRISPRi)	GGCTCATTGGCTCCACCCAG
sgRNA 5636 (CTCF - CRISPRi)	GACGTTCCCACTCTCCCTCC
sgRNA 5635 (CTCF - CRISPRi)	GGCACCACCTGGAGGGAGAG
sgRNA 15171 (CTCF - CRISPRa)	GAGTGACTGTCCTTCCACCA
sgRNA 15173 (CTCF - CRISPRa)	GTGACTGTCCTTCCACCAGG
sgRNA 13189 (CTCF - CRISPRa)	GTGTGGACGTGAGGGGGCAC
sgRNA 13190 (CTCF - CRISPRa)	GGCAGAATGTGGACGTGAGG
sgRNA 7138 (CTCF - CRISPRa)	GGTGAGCACCAGGAGGAGGG
sgRNA 7140 (CTCF - CRISPRa)	GGTGTGAGCACCAGGAGGAG
sgRNA 11698 (CTCF - CRISPRa)	GAGGACTCCAGGGCCCACAG
sgRNA 11699 (CTCF - CRISPRa)	GCTCCAGGGCCCACAGAGGG
sgRNA 16209 (CTCF - CRISPRa)	GGGCCCCTGGAGGCAGGAGT
sgRNA 16210 (CTCF - CRISPRa)	GGCCCAACTCCTGCCTCCAG
sgRNA 1S (Safe)	GCACATTTGGATTTCATGTC
sgRNA 1L (eGATA1)	GTTGGGGGAGACGAGGGCGG
sgRNA 2L (eGATA1)	GCAAGGAGGCAGCTGGGAGT
sgRNA 3L (eGATA1)	GACGGGGATGGGGGAGGGAA
sgRNA 1H (eGATA1)	GCGGGGTTTCCAGCTCTTGC
sgRNA 4L (eHDAC6)	GGCGGCAGGACATCTTCAAG
sgRNA 5L (eHDAC6)	GGGGAGTTGCGGGGGAGAGG
sgRNA 2H (eHDAC6)	GACACTTTCTATTACTGCTT
sgRNA N4293 (Safe)	GAGGAGAGCCAATGATCTCT
sgRNA 28310 (TSS)	GGTGATCCCAGGGGGTGTCC
sgRNA 28360 (TSS +500 bp)	GTAGAGCAGATAAGGGGTTT

sgRNA 28121 (eGATA1)	GGCGCCCTACTCCTCACCCT
sgRNA 28105 (eGATA1)	GGAATCAGTGGGGCCAGATC
sgRNA 28309 (eHDAC6)	GTGTGGCTGGGCCGAGAGCG
sgRNA 29249 (eHDAC6)	GAGATAGGTTCACGTAGGAG
Fwd primer ICE (eGATA1)	GGCAAACGCTTGACTCCTTA
Rev primer ICE (eGATA1)	CTCCTTGTGGTGCAAGTGTG
Fwd primer ICE (eHDAC6)	GCCTGCAAAGCCTAAGATGT
Rev primer ICE (eHDAC6)	AGGCATCGTTGTAAACCACA
sgRNA-mCherry lentiviral plasmid pMCB320 (https://www.addgene.org/89359/)	gcttaagcggtcgacggatcgggagatctcccgatcccctatggtgcactctcagtacaatctgctctgatgccgcatagttaagccagtatct<br>gctccctgcttgtgtgttggaggtcgctgagtagtgcgcgagcaaaatttaagctacaacaaggcaaggcttgaccgacaattgcatgaaga<br>atctgcttagggttaggcgttttgcgctgcttcgcgatgtacgggccagatatacgcgttgacattgattattgactagttattaatagtaatcaatta<br>cggggtcattagttcatagcccatatatggagttccgcgttacataacttacggtaaatggcccgcctggctgaccgcccaacgacccccgcc<br>cattgacgtcaataatgacgtatgttcccatagtaacgccaatagggactttccattgacgtcaatgggtggagtatttacggtaaactgcccac<br>ttggcagtacatcaagtgtatcatatgccaagtacgccccctattgacgtcaatgacggtaaatggcccgcctggcattatgcccagtacatg<br>accttatgggactttcctacttggcagtacatctacgtattagtcatcgctattaccatggtgatgcggttttggcagtacatcaatgggcgtggat<br>agcggtttgactcacggggatttccaagtctccaccccattgacgtcaatgggagtttgttttggcaccaaaatcaacgggactttccaaaatgt<br>cgtaacaactccgccccattgacgcaaatgggcggtaggcgtgtacggtgggaggtctatataagcagcgcgttttgcctgtactgggtctct<br>ctggttagaccagatctgagcctgggagctctctggctaactagggaacccactgcttaagcctcaataaagcttgccttgagtgcttcaagta<br>gtgtgtgcccgtctgttgtgtgactctggtaactagagatccctcagacccttttagtcagtgtggaaaatctctagcagtggcgcccgaacagg<br>gacttgaaagcgaaagggaaaccagaggagctctctcgacgcaggactcggcttgctgaagcgcgcacggcaagaggcgaggggcg<br>gcgactggtgagtacgccaaaaattttgactagcggaggctagaaggagagagatgggtgcgagagcgtcagtattaagcgggggaga<br>attagatcgcgatgggaaaaaattcggttaaggccagggggaaagaaaaaatataaattaaaacatatagtatgggcaagcagggagct<br>agaacgattcgcagttaatcctggcctgttagaaacatcagaaggctgtagacaaatactgggacagctacaaccatcccttcagacagga<br>tcagaagaacttagatcattatataatacagtagcaaccctctattgtgtgcatcaaaggatagagataaaagacaccaaggaagctttaga<br>caagatagaggaagagcaaaacaaaagtaagaccaccgcacagcaagcggccggccgcgctgatcttcagacctggaggaggagat<br>atgagggacaattggagaagtgaattatataaatataaagtagtaaaaattgaaccattaggagtagcacccaccaaggcaaagagaag<br>agtggtgcagagagaaaaaagagcagtgggaataggagctttgttccttgggttcttgggagcagcaggaagcactatgggcgcagcgtc<br>aatgacgctgacggtacaggccagacaattattgtctggtatagtgcagcagcagaacaatttgctgagggctattgaggcgcaacagcat<br>ctgttgcaactcacagtctggggcatcaagcagctccaggcaagaatcctggctgtggaaagatacctaaaggatcaacagctcctgggg<br>atttggggttgctctggaaaactcatttgcaccactgctgtgccttggaatgctagttggagtaataaatctctggaacagatttggaatcacacg<br>acctggatggagtgggacagagaaattaacaattacacaagcttaatacactccttaattgaagaatcgcaaaaccagcaagaaaagaat<br>gaacaagaattattggaattagataaatgggcaagtttgtggaattggtttaacataacaaattggctgtggtatataaaattattcataatgata<br>gtaggaggcttggtaggtttaagaatagtttttgctgtactttctatagtgaatagagttaggcagggatattcaccattatcgtttcagacccacct<br>cccaaccccgaggggacccgacaggcccgaaggaatagaagaagaaggtggagagagagacagagacagatccattcgattagtg<br>aacggatcggcactgcgtgcgccaattctgcagacaaatggcagtattcatccacaattttaaaagaaaaggggggattggggggtacagt<br>gcaggggaaagaatagtagacataatagcaacagacatacaaactaaagaattacaaaaacaaattacaaaaattcaaaattttcgggtt<br>tattacagggacagcagagatccagtttggttagtaccgggcccgctctagagatccgacgcgccatctctaggcccgcgccggcccccctc<br>gcacggacttgtgggagaagctcggctactcccctgccccggttaatttgcatataatatttcctagtaactatagaggcttaatgtgcgataaa<br>agacagataatctgttctttttaatactagctacattttacatgataggcttggatttctataacttcgtatagcatacattatacgaagttataaaca<br>gcacaaaaggaaactcaccctaactgtaaagtaattgtgtgttttgagactataaGtatcccttggagaaCCAcctTGTTGGACCAG<br>GATGGGCACCACCCGTTTAAGAGCTAAGCTGGAAACAGCATAGCAAGTTTAAATAAGGCTAGTC<br>CGTTATCAACTTGAAAAAGTGGCACCGAGTCGGTGCTTTTTTTCtcgagtactaggatccattaggcggccgc<br>gtggataaccgtattaccgccatgcatGTGCCCGTCAGTGGGCAGAGCGCACATCGCCCACAGTCCCCGAG<br>AAGTTGGGGGGAGGGGTCGGCAATTGAACCGGTGCCTAGAGAAGGTGGCGCGGGGTAAACT<br>GGGAAAGTGATGTCGTGTACTGGCTCCGCCTTTTTCCCGAGGGTGGGGGAGAACCGTATATAA<br>GTGCAGTAGTCGCCGTGAACGTTCTTTTTCGCAACGGGTTTGCCGCCAGAACACAGGTAAGTG<br>CCGTGTGTGGTTCCCGCGGGCCTGGCCTCTTTACGGGTTATGGCCCTTGCGTGCCTTGAATTA<br>CTTCCACCTGGCTGCAGTACGTGATTCTTGATCCCGAGCTTCGGGTTGGAAGTGGGTGGGAGA<br>GTTCGAGGCCTTGCGCTTAAGGAGCCCCTTCGCCTCGTGCTTGAGTTGAGGCCTGGCCTGGG<br>CGCTGGGGCCGCCGCGTGCGAATCTGGTGGCACCTTCGCGCCTGTCTCGCTGCTTTCGATAA<br>GTCTCTAGCCATTTAAAATTTTTGATGACCTGCTGCGACGCTTTTTTTCTGGCAAGATAGTCTTG<br>TAAATGCGGGCCAAGATCTGCACACTGGTATTTCGGTTTTTGGGGCCGCGGGCGGCGACGGG<br>GCCCGTGCGTCCCAGCGCACATGTTCGGCGAGGCGGGGCCTGCGAGCGCGGCCACCGAGAA<br>TCGGACGGGGGTAGTCTCAAGCTGGCCGGCCTGCTCTGGTGCCTGGCCTCGCGCCGCCGTGT<br>ATCGCCCCGCCCTGGGCGGCAAGGCTGGCCCGGTCGGCACCAGTTGCGTGAGCGGAAAGAT<br>GGCCGCTTCCCGGCCCTGCTGCAGGGAGCTCAAAATGGAGGACGCGGCGCTCGGGAGAGCG<br>GGCGGGTGAGTCACCCACACAAAGGAAAAGGGCCTTTCCGTCCTCAGCCGTCGCTTCATGTGA<br>CTCCACGGAGTACCGGGCGCCGTCCAGGCACCTCGATTAGTTCTCGcGCTTTTGGAGTACGTC<br>GTCTTTAGGTTGGGGGGAGGGGTTTTATGCGATGGAGTTTCCCCACACTGAGTGGGTGGAGAC<br>TGAAGTTAGGCCAGCTTGGCACTTGATGTAATTCTCCTTGGAATTTGCCCTTTTTGAGTTTGGAT<br>CTTGGTTCATTCTCAAGCCTCAGACAGTGGTTCAAAGTTTTTTTCTTCCATTTCAGGTGTCGTGA<br>gCTAGCCTAGCCCACCATGACCGAGTACAAGCCCACGGTGCGCCTCGCCACCCGCGACGACG<br>TCCCCCGGGCCGTACGCACCCTCGCCGCCGCGTTCGCCGACTACCCCGCCACGCGCCACAC<br>CGTCGACCCGGACCGCCACATCGAGCGGGTCACCGAGCTGCAAGAACTCTTCCTCACGCGCG<br>TCGGGCTCGACATCGGCAAGGTGTGGGTCGCGGACGACGGCGCCGCGGTGGCGGTCTGGAC<br>CACGCCGGAGAGCGTCGAAGCGGGGGCGGTGTTCGCCGAGATCGGCCCGCGCATGGCCGAG<br>TTGAGCGGTTCCCGGCTGGCCGCGCAGCAACAGATGGAAGGCCTCCTGGCGCCGCACCGGC<br>CCAAGGAGCCCGCGTGGTTCCTGGCCACCGTCGGCGTCTCGCCCGACCACCAGGGCAAGGG<br>TCTGGGCAGCGCCGTCGTGCTCCCCGGAGTGGAGGCGGCCGAGCGCGCCGGGGTGCCCGC<br>CTTCCTGGAGACCTCCGCGCCCCGCAACCTCCCCTTCTACGAGCGGCTCGGCTTCACCGTCAC<br>CGCCGACGTCGAGGTGCCCGAAGGACCGCGCACCTGGTGCATGACCCGCAAGCCCGGTGCC

	GGATCGGGAGAGGGCAGAGGAAGTCTGCTAACATGCGGTGACGTCGAGGAGAATCCTGGCCC<br>Accggtcgccaccatggtgagcaagggcgaggaggataacatggccatcatcaaggagttcatgcgcttcaaggtgcacatggagggct<br>ccgtgaacggccacgagttcgagatcgagggcgagggcgagggccgcccctacgagggcacccagaccgccaagctgaaggtgacc<br>aagggtggcccctgcccttcgcctgggacatcctgtcccctcagttcatgtacggctccaaggcctacgtgaagcaccccgccgacatccc<br>cgactacttgaagctgtccttccccgagggcttcaagtgggagcgcgtgatgaacttcgaggacggcggcgtggtgaccgtgacccagga<br>ctcctccctgcaggacggcgagttcatctacaaggtgaagctgcgcggcaccaacttcccctccgacggccccgtaatgcagaagaagac<br>catgggctgggaggcctcctccgagcggatgtaccccgaggacggcgccctgaagggcgagatcaagcagaggctgaagctgaagga<br>cggcggccactacgacgctgaggtcaagaccacctacaaggccaagaagcccgtgcagctgcccggcgcctacaacgtcaacatcaa<br>gttggacatcacctcccacaacgaggactacaccatcgtggaacagtacgaacgcgccgagggccgccactccaccggcggcatggac<br>gagctgtacaagtaagagaattcgtcgagggacctaataacttcgtatagcatacattatacgaagttatacatgtttaagggttccggttccac<br>taggtacaattcgatatcaagcttatcgataatcaacctctggattacaaaatttgtgaaagattgactggtattcttaactatgttgctccttttacg<br>ctatgtggatacgctgctttaatgcctttgtatcatgctattgcttcccgtatggctttcattttctcctccttgtataaatcctggttgctgtctctttatgag<br>gagttgtggcccgttgtcaggcaacgtggcgtggtgtgcactgtgtttgctgacgcaacccccactggttggggcattgccaccacctgtcagc<br>tcctttccgggactttcgctttccccctccctattgccacggcggaactcatcgccgcctgccttgcccgctgctggacaggggctcggctgttgg<br>gcactgacaattccgtggtgttgtcggggaaatcatcgtcctttccttggctgctcgcctgtgttgccacctggattctgcgcgggacgtccttctg<br>ctacgtcccttcggccctcaatccagcggaccttccttcccgcggcctgctgccggctctgcggcctcttccgcgtcttcgccttcgccctcaga<br>cgagtcggatctccctttgggccgcctccccgcatcgataccgtcgacctcgatcgagacctagaaaaacatggagcaatcacaagtagc<br>aatacagcagctaccaatgctgattgtgcctggctagaagcacaagaggaggaggaggtgggttttccagtcacacctcaggtacctttaa<br>gaccaatgacttacaaggcagctgtagatcttagccactttttaaaagaaaaggggggactggaagggctaattcactcccaacgaagac<br>aagatatccttgatctgtggatctaccacacacaaggctacttccctgattggcagaactacacaccagggccagggatcagatatccactg<br>acctttggatggtgctacaagctagtaccagttgagcaagagaaggtagaagaagccaatgaaggagagaacacccgcttgttacaccct<br>gtgagcctgcatgggatggatgacccggagagagaagtattagagtggaggtttgacagccgcctagcatttcatcacatggcccgagag<br>ctgcatccggactgtactgggtctctctggttagaccagatctgagcctgggagctctctggctaactagggaacccactgcttaagcctcaat<br>aaagcttgccttgagtgcttcaagtagtgtgtgcccgtctgttgtgtgactctggtaactagagatccctcagacccttttagtcagtgtggaaaat<br>ctctagcagcatgtgagcaaaaggccagcaaaaggccaggaaccgtaaaaaggccgcgttgctggcgtttttccataggctccgcccccc<br>tgacgagcatcacaaaaatcgacgctcaagtcagaggtggcgaaacccgacaggactataaagataccaggcgtttccccctggaagct<br>ccctcgtgcgctctcctgttccgaccctgccgcttaccggatacctgtccgcctttctcccttcgggaagcgtggcgctttctcatagctcacgctg<br>taggtatctcagttcggtgtaggtcgttcgctccaagctgggctgtgtgcacgaaccccccgttcagcccgaccgctgcgccttatccggtaact<br>atcgtcttgagtccaacccggtaagacacgacttatcgccactggcagcagccactggtaacaggattagcagagcgaggtatgtaggcg<br>gtgctacagagttcttgaagtggtggcctaactacggctacactagaagaacagtatttggtatctgcgctctgctgaagccagttaccttcgga<br>aaaagagttggtagctcttgatccggcaaacaaaccaccgctggtagcggtggtttttttgtttgcaagcagcagattacgcgcagaaaaaa<br>aggatctcaagaagatcctttgatcttttctacggggtctgacgctcagtggaacgaaaactcacgttaagggattttggtcatgagattatcaa<br>aaaggatcttcacctagatccttttaaattaaaaatgaagttttaaatcaatctaaagtatatatgagtaaacttggtctgacagttaccaatgctt<br>aatcagtgaggcacctatctcagcgatctgtctatttcgttcatccatagttgcctgactccccgtcgtgtagataactacgatacgggagggctt<br>accatctggccccagtgctgcaatgataccgcgagacccacgctcaccggctccagatttatcagcaataaaccagccagccggaaggg<br>ccgagcgcagaagtggtcctgcaactttatccgcctccatccagtctattaattgttgccgggaagctagagtaagtagttcgccagttaatagt<br>ttgcgcaacgttgttgccattgctacaggcatcgtggtgtcacgctcgtcgtttggtatggcttcattcagctccggttcccaacgatcaaggcga<br>gttacatgatcccccatgttgtgcaaaaaagcggttagctccttcggtcctccgatcgttgtcagaagtaagttggccgcagtgttatcactcatg<br>gttatggcagcactgcataattctcttactgtcatgccatccgtaagatgcttttctgtgactggtgagtactcaaccaagtcattctgagaatagtg<br>tatgcggcgaccgagttgctcttgcccggcgtcaatacgggataataccgcgccacatagcagaactttaaaagtgctcatcattggaaaac<br>gttcttcggggcgaaaactctcaaggatcttaccgctgttgagatccagttcgatgtaacccactcgtgcacccaactgatcttcagcatcttttta<br>ctttcaccagcgtttctgggtgagcaaaaacaggaaggcaaaatgccgcaaaaaagggaataagggcgacacggaaatgttgaatactc<br>atactcttcctttttcaatattattgaagcatttatcagggttattgtctcatgagcggatacatatttgaatgtatttagaaaaataaacaaataggg<br>gttccgcgcacatttccccgaaaagtgccacctgac

Supplementary Table 2: Mapping and QC statistics for ChIP-seq datasets used in this study

Dataset	Library complexity	NSC	RSC	QC	Read Length	Mapped reads	Raw fragments
L111-Cas9-sgRNA-N4293-Input	0.96	1.121	0.303	-1	2x75	84,490,748	55,837,397
L120-Cas9-sgRNA-N4293-CTCF	0.93	4.111	1.563	2	2x75	34,775,812	23,853,267
L121-Cas9-sgRNA-8005-CTCF	0.9	8.302	1.909	2	2x75	31,881,148	22,583,697
L122-Cas9-sgRNA-12040-CTCF	0.91	8.584	1.827	2	2x75	20,212,130	14,111,997
L123-Cas9-sgRNA-13004-CTCF	0.87	8.154	1.905	2	2x75	27,329,576	19,091,732
L124-Cas9-sgRNA-14259-CTCF	0.91	9.061	1.932	2	2x75	30,866,786	21,744,890
L125-Cas9-sgRNA-14376-CTCF	0.92	9.269	1.874	2	2x75	26,398,240	19,111,658
L126-Cas9-sgRNA-15776-CTCF	0.91	10.139	1.953	2	2x75	29,279,448	20,919,316

Supplementary Table 3: Mapping and QC statistics for RNA-seq datasets used in this study

Library	Raw fragments	Complexity	Unique	Unique Splices	Multi	Multi Splices	Fraction mapped
12040_1_CTCFvalidation_S1	28,644,781	0.68	20,358,952	4,132,800	2,424,064	640,956	0.48
12040_2_CTCFvalidation_S2	28,846,455	0.67	19,606,123	3,975,313	2,319,439	621,135	0.46
12042_1_CTCFvalidation_S3	36,357,512	0.66	25,770,216	5,389,554	3,016,467	830,405	0.48
12042_2_CTCFvalidation_S4	32,696,948	0.67	22,560,933	4,604,545	2,604,102	697,272	0.47
13004_1_CTCFvalidation_S9	28,892,443	0.68	20,776,068	4,282,772	2,438,370	638,984	0.49
13004_2_CTCFvalidation_S10	28,460,588	0.68	19,421,721	3,951,549	2,276,571	609,239	0.46
13006_1_CTCFvalidation_S11	35,962,940	0.66	25,727,017	5,352,693	3,000,594	821,216	0.49
13006_2_CTCFvalidation_S12	31,497,868	0.68	20,750,328	4,296,768	2,458,910	666,244	0.45
14376_1_CTCFvalidation_S5	38,779,897	0.66	27,758,193	5,792,893	3,271,621	888,843	0.49
14376_2_CTCFvalidation_S6	28,806,065	0.68	18,814,700	3,832,676	2,155,832	583,804	0.44
14377_1_CTCFvalidation_S7	30,361,030	0.68	21,620,017	4,310,279	2,522,358	664,134	0.48
14377_2_CTCFvalidation_S8	36,713,802	0.66	24,774,171	5,079,087	2,940,671	801,139	0.46
N4293_1_CTCFvalidation_S13	31,819,345	0.67	22,969,926	4,745,722	2,665,517	724,855	0.49
N4293_2_CTCFvalidation_S14	31,045,772	0.68	20,376,549	4,265,365	2,382,260	649,440	0.45
N4871_1_CTCFvalidation_S15	33,515,599	0.65	24,289,758	5,053,530	2,865,219	765,455	0.49
N4871_2_CTCFvalidation_S16	19,600,752	0.72	13,567,179	2,753,987	1,553,040	416,586	0.47

Supplementary Table 4: Mapping and QC statistics for ATAC-seq datasets used in this study

Library	Raw fragments	Unique reads	Complexity	chrM reads	chrM fraction	Unique non-chrM reads after dedup	TSS ratio	MACS default peaks	FRiP (MACS)	post IDR (0.05) peaks, ind. Reps	FRIP (IDR)
Cas9-13004-1	52,789,893	61,048,866	0.82	33,514,562	0.32	50,711,620	14.36	60,654	0.21	43,160	0.22
Cas9-13004-2	18,231,944	21,188,935	0.87	12,106,612	0.33	18,580,257	14.85	30,715	0.17	43,160	0.22
Cas9-13006-1	28,470,155	31,019,550	0.87	19,482,884	0.34	27,179,398	15.3	38,581	0.19	42,584	0.23
Cas9-13006-2	21,881,230	24,333,878	0.86	15,785,470	0.36	21,233,756	16.12	36,687	0.19	42,584	0.24
Cas9-N4293-1	27,588,151	35,157,791	0.85	13,635,924	0.25	30,100,001	12.97	47,341	0.16	43,500	0.19
Cas9-N4293-2	19,786,147	24,311,620	0.86	11,934,332	0.3	21,179,734	16.86	39,213	0.21	43,500	0.25
Cas9-N4371-1	30,394,942	36,688,985	0.84	17,928,778	0.29	31,230,415	16.19	48,875	0.22	50,962	0.25
Cas9-N4371-2	23,086,373	27,737,031	0.85	14,534,148	0.31	24,025,735	17.09	45,001	0.22	50,962	0.27

Supplementary Methods

sgRNA targeting CTCF motif library design

In addition to the 4,022 canonical loop anchor CTCF binding sites (Type 0), we added to the screen a small set of 310 sites (Types 1 - 5) that would allow us to test additional hypotheses about the role of CTCF in gene regulation and genome 3D architecture. For each hypothesis, we started with 100 candidate sites, and as before, filtered out the ones with ≥2 sgRNAs passing filtering criteria. However, upon discovering the dominant effect of confounding off-target activity in the CTCF motif screen, which was similarly dominant among sites of Types 1 - 5, we decided not to include these additional types in the analyses and figures for the sake of clarity.

Type 1: Loop anchors without annotated CTCF binding sites annotated using binding preferences obtained from deep learning models for predicting TF binding: We hypothesized that we could expand the set of binding loop anchors tested by including CTCF sites that fall below the motif-calling threshold used for annotation before [12] but for which formation of the loop might still be occurring through a CTCF-mediated mechanism. We required remaining unannotated loop anchors to be in a TAD with genes with strong growth effects in gene-level knockout screens. Then, within the loop anchors, we annotated likely CTCF “motifs” by identifying subsequences with high importance for binding in a TF binding prediction deep learning model (described below, **Supplementary Materials & Methods**). Importance scores were derived using DeepLIFT with gradient times input [13]; important subsequences were defined as those that for which the cross-correlation of the DeepLIFT gradient times input scores and the CTCF JASPAR motif [14] was large. After guide filtering, we were left with 80 sites.

Type 2: Rad21 ChIP-exo peaks in TADs with strongest growth genes: Here we asked whether the growth effects we might observe at CTCF binding sites are due to disruption of CTCF binding, disruption of RAD21 (or more generally the cohesin complex) function or both. Previous work that used ChIP-exo to map the precise binding of CTCF and RAD21 suggested that these 2 proteins occur in specific spacing and orientations [15], allowing us to test this hypothesis. Thus, to identify RAD21-specific binding sites, we used the existing data from that work as follows. Since Tang et al. did not profile ChIP-exo of RAD21 in K562 cells, we started from the ChIP-exo RAD21 sites measured in GM12878 cells. We kept the RAD21 ChIP-exo sites that overlapped ChIP-seq peaks for RAD21 in K562 cells and required them to be within 100 bp to the right of the CTCF

motifs from our screen, consistent with the positioning of RAD21 ChIP-exo sites relative to CTCF ChIP-exo sites in Tang et al. We also prioritized RAD21 binding sites annotated as GSB (spikes on both sides of the binding sites), which are more confident ChIP-exo calls. After filtering, we obtained 72 such sites.

Type 4: CTCF ChIP-seq peaks outside loop anchors within DNase hypersensitive regions: In order to compare loop-anchor CTCF sites to non-loop anchor CTCF sites, we selected CTCF sites from the latter category within the top 100 TADs containing growth genes subject to the requirement that they overlap K562 DNase hypersensitive regions from the Roadmap Epigenomics data from the University of Washington [16]. We then defined the precise CTCF binding site based on DeepLIFT scores as described above. The final set after filtering contained 82 sites.

Type 5: CTCF ChIP-seq peaks outside loop anchors outside DNase hypersensitive sites: This category was defined as above except that CTCF sites were required to not overlap K562 DNase hypersensitive regions. The final set in this case consisted of 76 sites. For the analyses used here (**Figure 1**), we filtered the library to remove Types 1-5.

Deep learning models for TF binding prediction used for CTCF library design:

To select CTCF sites in the category of loop anchors without annotated CTCF binding sites, we trained a deep convolutional neural network (CNN) to predict whether a sequence is a CTCF binding site or an open chromatin region without CTCF, computed importance scores for nucleotides' importance for the model's predictions, and compared the nucleotides weighted by their importance scores to a PWM for CTCF. The positive set in CNN training consisted of the ±500bp sequences around IDR-reproducible black list-filtered ENCODE K562 CTCF ChIP-seq peak summits (ENCODE accession ID ENCSR000DMA) [17]. For the negative set, the ±500bp sequences around Epigenomic Roadmap [16] K562 DNase peak summits that did not overlap any K562 CTCF peaks (including non-reproducible peaks) were used. The training set consisted of chromosomes 3-7, 10-22 and X; the validation set (used for hyper-parameter tuning) consisted of chromosomes 8 and 9, and the test set consisted of chromosomes 1 and 2. Sequences were one-hot encoded as 4*1000 binary matrices following previously established practices [18,19]. "N" bases were encoded as zeros. We separately encoded each sequence and its reverse complement.

We used an architecture featuring three convolutional layers, with each followed by a rectified linear unit (ReLU), followed by a max-pooling layer. The convolutional filters of the first layer can be interpreted as picking up sequence patterns revealing whether a peak is a CTCF peak or a DNase peak without CTCF, the filters in the following layers identify combinations of those patterns, and the max-pooling layer encodes the assumption that a single sequence pattern combination should not occur multiple times within a short region. The first convolutional layer had sixty (4*15) filters with stride 1*1, the second convolutional layer had 60 (1*15) filters with stride 1*1, and the third convolutional layer had 15 (1*15) filters with stride 1*1. Each layer used a dropout rate of 0.2. The max-pooling layer was of size 1*35 and stride 1*35. The max-pooling layer was followed by a sigmoid. The model was trained using Keras version 0.3.2 [20] with stochastic gradient descent with Nesterov momentum 0.85, learning rate 0.01, and batch size 200. The model was trained for 47 epochs. Weights were initialized from a pre-trained model with the same hyper-parameters and the negative set randomly down-sampled to be the size of the positive set, where the model was trained for 100 epochs. Weights for pre-training were initialized using Keras's He normal initializer [20,21].

To identify regions within ChIP-seq peaks that are important for making positive predictions, we scored the importance of every nucleotide in each positive example using DeepLIFT with gradient times input, which computes the product of each input and gradient with respect to that input (Shrikumar, Greenside, & Kundaje, 2017). Since most CTCF ChIP-seq peaks that were correctly predicted had at least one region with high DeepLIFT scores and we wanted to select less than one hundred guides, we filtered the regions with high DeepLIFT scores by cross-correlating the scores starting at each index within the sequence with the log-odds of the CTCF PWM from JASPAR [14], where we used a pseudo-count of 0.0001 and a background of 52% GC content when computing the log-odds. This was procedure was carried out for each sequence and its reverse complement, and the top two motif hits across both were retained. (Note that some of these motif hits would not be identified by scanning the sequence for the CTCF motif because the regions with important DeepLIFT scores are not always those with the best matches to the CTCF motif.) Motif hits within 20 bp of a higher-scoring motif hit as well as those with log-odds scores ≤0.5 were removed. Motif hits in peaks without a previously identified CTCF motif hit were retained; the intuition is that these sequences are imperfect matches to the CTCF motif that are missed by PWM scanning.

Supplementary References

1. Bae, S., Park, J. & Kim, J.-S. Cas-OFFinder: a fast and versatile algorithm that searches for potential off-target sites of Cas9 RNA-guided endonucleases. *Bioinformatics* **30**, 1473–1475 (2014).
2. Morgens, D. W. *et al.* Genome-scale measurement of off-target activity using Cas9 toxicity in high-throughput screens. *Nat. Commun.* **8**, 15178 (2017).
3. Horlbeck, M. A. *et al.* Compact and highly active next-generation libraries for CRISPR-mediated gene repression and activation. *Elife* **5**, (2016).
4. Tsai, S. Q. *et al.* GUIDE-seq enables genome-wide profiling of off-target cleavage by CRISPR-Cas nucleases. *Nat. Biotechnol.* **33**, 187–198 (2015).
5. Hsu, P. D. *et al.* DNA targeting specificity of RNA-guided Cas9 nucleases. *Nat. Biotechnol.* **31**, 827–832 (2013).
6. Haeussler, M. *et al.* Evaluation of off-target and on-target scoring algorithms and integration into the guide RNA selection tool CRISPOR. *Genome Biol.* **17**, 148 (2016).
7. Morgens, D. W., Deans, R. M., Li, A. & Bassik, M. C. Systematic comparison of CRISPR/Cas9 and RNAi screens for essential genes. *Nat. Biotechnol.* **34**, 634–636 (2016).
8. Hsiau, T. *et al.* Inference of CRISPR Edits from Sanger Trace Data. *bioRxiv* 251082 (2018). doi:10.1101/251082
9. Brinkman, E. K., Chen, T., Amendola, M. & van Steensel, B. Easy quantitative assessment of genome editing by sequence trace decomposition. *Nucleic Acids Res.* **42**, e168 (2014).
10. Yeo, N. C. *et al.* An enhanced CRISPR repressor for targeted mammalian gene regulation. *Nat. Methods* **15**, 611–616 (2018).
11. Rosenbluh, J. *et al.* Complementary information derived from CRISPR Cas9 mediated gene deletion and suppression. *Nat. Commun.* **8**, 15403 (2017).

12. Rao, S. S. P. *et al.* A 3D map of the human genome at kilobase resolution reveals principles of chromatin looping. *Cell* **159**, 1665–1680 (2014).

13. Shrikumar, A., Greenside, P. & Kundaje, A. Reverse-complement parameter sharing improves deep learning models for genomics. *bioRxiv* 103663 (2017). doi:10.1101/103663

14. Mathelier, A. *et al.* JASPAR 2016: a major expansion and update of the open-access database of transcription factor binding profiles. *Nucleic Acids Res.* **44**, D110–5 (2016).

15. Tang, Z. *et al.* CTCF-Mediated Human 3D Genome Architecture Reveals Chromatin Topology for Transcription. *Cell* **163**, 1611–1627 (2015).

16. Roadmap Epigenomics Consortium *et al.* Integrative analysis of 111 reference human epigenomes. *Nature* **518**, 317–330 (2015).

17. Dunham, I. *et al.* An integrated encyclopedia of DNA elements in the human genome. *Nature* **489**, 57–74 (2012).

18. Alipanahi, B., Delong, A., Weirauch, M. T. & Frey, B. J. Predicting the sequence specificities of DNA- and RNA-binding proteins by deep learning. *Nat. Biotechnol.* **33**, 831–838 (2015).

19. Kelley, D. R., Snoek, J. & Rinn, J. L. Basset: learning the regulatory code of the accessible genome with deep convolutional neural networks. *Genome Res.* **26**, 990–999 (2016).

20. Chollet, F. & Others. Keras. (2015).

21. He, K., Zhang, X., Ren, S. & Sun, J. Delving Deep into Rectifiers: Surpassing Human-Level Performance on ImageNet Classification. *arXiv [cs.CV]* (2015).
