# Supplementary material for: Mitigation of off-target toxicity in CRISPR-Cas9 screens for essential non-coding elements
Source: Nat Commun. 2019 Sep 6;10:4063. doi: 10.1038/s41467-019-11955-7 (PMC6731277; doi:10.1038/s41467-019-11955-7)
Supplement: Supplementary file 2 — Reporting Summary [file 41467_2019_11955_MOESM2_ESM.pdf]

nature research

Corresponding author(s): Michael Bassik

Last updated by author(s): Jul 24, 2019

Reporting Summary

Nature Research wishes to improve the reproducibility of the work that we publish. This form provides structure for consistency and transparency in reporting. For further information on Nature Research policies, see Authors & Referees and the Editorial Policy Checklist.

Statistics

For all statistical analyses, confirm that the following items are present in the figure legend, table legend, main text, or Methods section.

n/a	Confirmed	
☐	☒	The exact sample size (*n*) for each experimental group/condition, given as a discrete number and unit of measurement
☐	☒	A statement on whether measurements were taken from distinct samples or whether the same sample was measured repeatedly
☐	☒	The statistical test(s) used AND whether they are one- or two-sided<br>*Only common tests should be described solely by name; describe more complex techniques in the Methods section.*
☐	☒	A description of all covariates tested
☐	☒	A description of any assumptions or corrections, such as tests of normality and adjustment for multiple comparisons
☐	☒	A full description of the statistical parameters including central tendency (e.g. means) or other basic estimates (e.g. regression coefficient) AND variation (e.g. standard deviation) or associated estimates of uncertainty (e.g. confidence intervals)
☐	☒	For null hypothesis testing, the test statistic (e.g. *F*, *t*, *r*) with confidence intervals, effect sizes, degrees of freedom and *P* value noted<br>*Give P values as exact values whenever suitable.*
☐	☒	For Bayesian analysis, information on the choice of priors and Markov chain Monte Carlo settings
☐	☒	For hierarchical and complex designs, identification of the appropriate level for tests and full reporting of outcomes
☐	☒	Estimates of effect sizes (e.g. Cohen's *d*, Pearson's *r*), indicating how they were calculated

Our web collection on statistics for biologists contains articles on many of the points above.

Software and code

Policy information about availability of computer code

Data collection	GuideScan v1.0 for sgRNA design.
Data analysis	For screen analysis we used custom Python scripts available at https://bitbucket.org/dmorgens/castle. Flowjo 9.9 and CytoFlow v0.9.3 were used for flow cytometry analyses. FIMO (version 4.12.0 of the MEME-Suite) for motif mapping. Bowtie v1.0.1 for read alignment. Total RNA-seq reads were mapped using version 2.5.3a of the STAR aligner. Differential expression analysis was performed using DESeq2 . ICE indel analysis (provided online by Synthego).<br>We also wrote custom Python scripts for 1) library design for non-coding screens with specificity filtering and 2) retrieval of GuideScan specificity scores for pre-existing guide libraries, which we make available via GitHub (https://github.com/georgimarinov/non_coding_CRISPR_screen_design).

For manuscripts utilizing custom algorithms or software that are central to the research but not yet described in published literature, software must be made available to editors/reviewers. We strongly encourage code deposition in a community repository (e.g. GitHub). See the Nature Research guidelines for submitting code & software for further information.

Data

Policy information about availability of data

All manuscripts must include a data availability statement. This statement should provide the following information, where applicable:

- Accession codes, unique identifiers, or web links for publicly available datasets
- A list of figures that have associated raw data
- A description of any restrictions on data availability

The following datasets are accessible in the online GEO repository with accession GSE131349: CRISPR-Cas9 screen data (tiling screens, dense-tiling screen, CTCF motif screens), CTCF ChIP-seq, ATAC-seq, RNA-seq [https://www.ncbi.nlm.nih.gov/geo/query/acc.cgi?acc=GSE131349].

Field-specific reporting

Please select the one below that is the best fit for your research. If you are not sure, read the appropriate sections before making your selection.

☒ Life sciences ☐ Behavioural & social sciences ☐ Ecological, evolutionary & environmental sciences

For a reference copy of the document with all sections, see nature.com/documents/nr-reporting-summary-flat.pdf

Life sciences study design

All studies must disclose on these points even when the disclosure is negative.

Sample size	Not done
Data exclusions	As described in the Supplementary Information, a small set of 310 non-Type 0 loop anchor target sites (Types 1 - 5) were included in the CTCF motif screen but not included in the analyses and figures for the sake of clarity, upon the discovery of the dominant effect of confounding off-target activity, which similarly impacted the Types 1 - 5 sgRNAs.
Replication	Once experiments and procedures were fully optimized, all attempts at replication were successful. To ensure reproducibility every experiment performed in replicate. Number of replicates for each experiment is indicated in figure legends.
Randomization	Not done
Blinding	Not done

Reporting for specific materials, systems and methods

We require information from authors about some types of materials, experimental systems and methods used in many studies. Here, indicate whether each material, system or method listed is relevant to your study. If you are not sure if a list item applies to your research, read the appropriate section before selecting a response.

Materials & experimental systems

n/a	Involved in the study	
☐	☒	Antibodies
☐	☒	Eukaryotic cell lines
☐	☐	Palaeontology
☐	☐	Animals and other organisms
☐	☐	Human research participants
☐	☐	Clinical data

Methods

n/a	Involved in the study	
☐	☒	ChIP-seq
☐	☒	Flow cytometry
☐	☐	MRI-based neuroimaging

Antibodies

Antibodies used	polyclonal anti-CTCF antibody (Millipore, 07-729)
Validation	Website states: "Routinely evaluated by Western Blot.<br><br>Western Blot Analysis:<br>A 1:1000–1:5000 dilution of this lot detected CTCF in HeLa nuclear extract. A previous lot detected CTCF in K562 nuclear extract (data not shown)."

Eukaryotic cell lines

Policy information about cell lines

Cell line source(s)	K562 (ATCC CCL-243)
Authentication	Cell lines were authenticated by the vendor (ATCC). Authentication includes an assay to detect species specific variants of the cytochrome C oxidase I gene (COI analysis) to rule out inter-species contamination and short tandem repeat (STR) profiling to distinguish between individual human cell lines and rule out intra-species contamination.
Mycoplasma contamination	Cell cultures were routinely tested and found negative for mycoplasma infection (MycoAlert, Lonza).

Commonly misidentified lines (See ICLAC register)	None of the cell lines used in this study are in the database of commonly misidentified cell lines.

Palaeontology

Specimen provenance	*Provide provenance information for specimens and describe permits that were obtained for the work (including the name of the issuing authority, the date of issue, and any identifying information).*
Specimen deposition	*Indicate where the specimens have been deposited to permit free access by other researchers.*
Dating methods	*If new dates are provided, describe how they were obtained (e.g. collection, storage, sample pretreatment and measurement), where they were obtained (i.e. lab name), the calibration program and the protocol for quality assurance OR state that no new dates are provided.*

☐ Tick this box to confirm that the raw and calibrated dates are available in the paper or in Supplementary Information.

Animals and other organisms

Policy information about studies involving animals; ARRIVE guidelines recommended for reporting animal research

Laboratory animals	*For laboratory animals, report species, strain, sex and age OR state that the study did not involve laboratory animals.*
Wild animals	*Provide details on animals observed in or captured in the field; report species, sex and age where possible. Describe how animals were caught and transported and what happened to captive animals after the study (if killed, explain why and describe method; if released, say where and when) OR state that the study did not involve wild animals.*
Field-collected samples	*For laboratory work with field-collected samples, describe all relevant parameters such as housing, maintenance, temperature, photoperiod and end-of-experiment protocol OR state that the study did not involve samples collected from the field.*
Ethics oversight	*Identify the organization(s) that approved or provided guidance on the study protocol, OR state that no ethical approval or guidance was required and explain why not.*

Note that full information on the approval of the study protocol must also be provided in the manuscript.

Human research participants

Policy information about studies involving human research participants

Population characteristics	*Describe the covariate-relevant population characteristics of the human research participants (e.g. age, gender, genotypic information, past and current diagnosis and treatment categories). If you filled out the behavioural & social sciences study design questions and have nothing to add here, write "See above."*
Recruitment	*Describe how participants were recruited. Outline any potential self-selection bias or other biases that may be present and how these are likely to impact results.*
Ethics oversight	*Identify the organization(s) that approved the study protocol.*

Note that full information on the approval of the study protocol must also be provided in the manuscript.

Clinical data

Policy information about clinical studies

All manuscripts should comply with the ICMJE guidelines for publication of clinical research and a completed CONSORT checklist must be included with all submissions.

Clinical trial registration	*Provide the trial registration number from ClinicalTrials.gov or an equivalent agency.*
Study protocol	*Note where the full trial protocol can be accessed OR if not available, explain why.*
Data collection	*Describe the settings and locales of data collection, noting the time periods of recruitment and data collection.*
Outcomes	*Describe how you pre-defined primary and secondary outcome measures and how you assessed these measures.*

ChIP-seq

Data deposition

☒ Confirm that both raw and final processed data have been deposited in a public database such as GEO.

☒ Confirm that you have deposited or provided access to graph files (e.g. BED files) for the called peaks.

Data access links
May remain private before publication.

[https://www.ncbi.nlm.nih.gov/geo/query/acc.cgi?acc=GSE131349]

Files in database submission

Provide a list of all files available in the database submission.

Genome browser session
(e.g. UCSC)

Provide a link to an anonymized genome browser session for "Initial submission" and "Revised version" documents only, to enable peer review. Write "no longer applicable" for "Final submission" documents.

Methodology

Replicates

Two biological replicates, separately transduced with sgRNAs, were grown and then subjected to ChIP-seq.

Sequencing depth

Provided in a supplementary table.

Antibodies

polyclonal anti-CTCF antibody (Millipore, 07-729)

Peak calling parameters

As described in Methods:
For analyses of CTCF occupancy, reads were mapped against the female version of the hg19 assembly of the human genome using the 'bwa mem' algorithm in the BWA aligner with default settings and filtering non-unique and low-quality alignments using samtools 85 with the '-F 180 -q 30' options. A consensus set of peaks was derived from the three "safe" sgRNA CTCF ChIP-seq datasets as described in the AQUAS pipeline. FRiP values 95 were calculated for each dataset using this set of peak calls. Read coverage tracks were generated using custom-written Python scripts.

Data quality

Demultipexed fastq files were initially mapped to the hg19 assembly of the human genome (female version) as 1x36mers using Bowtie v1.0.1 94 with the following settings: '-v 2 -k 2 -m 1 --best --strata', for quality assessment purposes (see AQUAS: https://github.com/kundajelab/chipseq_pipeline) (Supplementary Table 3).

Software

AQUAS: https://github.com/kundajelab/chipseq_pipeline

Flow Cytometry

Plots

Confirm that:

☒ The axis labels state the marker and fluorochrome used (e.g. CD4-FITC).

☒ The axis scales are clearly visible. Include numbers along axes only for bottom left plot of group (a 'group' is an analysis of identical markers).

☐ All plots are contour plots with outliers or pseudocolor plots.

☐ A numerical value for number of cells or percentage (with statistics) is provided.

Methodology

Sample preparation

K562 suspension cells were transferred from flasks or plates into flow tubes and subjected to flow cytometry.

Instrument

BD FACSAria II, BD Accuri C6, and Beckman Coulter CytoFLEX instruments were used.

Software

Flowjo 9.9 and CytoFlow v0.9.3 were used for flow cytometry analyses.

Cell population abundance

Our samples were mixtures of K562 expressing an sgRNA and mCherry with blank parental K562 cells, as described in the Methods.

Gating strategy

First, a polygonal gate was drawn on FSC/SSC to isolate single viable cells, then a linear gate was drawn between the mCherry positive and negative cells.

☐ Tick this box to confirm that a figure exemplifying the gating strategy is provided in the Supplementary Information.

Magnetic resonance imaging

Experimental design

Design type

Indicate task or resting state; event-related or block design.

Design specifications

Specify the number of blocks, trials or experimental units per session and/or subject, and specify the length of each trial or block (if trials are blocked) and interval between trials.

Behavioral performance measures

State number and/or type of variables recorded (e.g. correct button press, response time) and what statistics were used to establish that the subjects were performing the task as expected (e.g. mean, range, and/or standard deviation across subjects).

Acquisition

Imaging type(s)	*Specify: functional, structural, diffusion, perfusion.*
Field strength	*Specify in Tesla*
Sequence & imaging parameters	*Specify the pulse sequence type (gradient echo, spin echo, etc.), imaging type (EPI, spiral, etc.), field of view, matrix size, slice thickness, orientation and TE/TR/flip angle.*
Area of acquisition	*State whether a whole brain scan was used OR define the area of acquisition, describing how the region was determined.*

Diffusion MRI ☐ Used ☐ Not used

Preprocessing

Preprocessing software	*Provide detail on software version and revision number and on specific parameters (model/functions, brain extraction, segmentation, smoothing kernel size, etc.).*
Normalization	*If data were normalized/standardized, describe the approach(es): specify linear or non-linear and define image types used for transformation OR indicate that data were not normalized and explain rationale for lack of normalization.*
Normalization template	*Describe the template used for normalization/transformation, specifying subject space or group standardized space (e.g. original Talairach, MNI305, ICBM152) OR indicate that the data were not normalized.*
Noise and artifact removal	*Describe your procedure(s) for artifact and structured noise removal, specifying motion parameters, tissue signals and physiological signals (heart rate, respiration).*
Volume censoring	*Define your software and/or method and criteria for volume censoring, and state the extent of such censoring.*

Statistical modeling & inference

Model type and settings	*Specify type (mass univariate, multivariate, RSA, predictive, etc.) and describe essential details of the model at the first and second levels (e.g. fixed, random or mixed effects; drift or auto-correlation).*
Effect(s) tested	*Define precise effect in terms of the task or stimulus conditions instead of psychological concepts and indicate whether ANOVA or factorial designs were used.*

Specify type of analysis: ☐ Whole brain ☐ ROI-based ☐ Both

Statistic type for inference (See Eklund et al. 2016)	*Specify voxel-wise or cluster-wise and report all relevant parameters for cluster-wise methods.*
Correction	*Describe the type of correction and how it is obtained for multiple comparisons (e.g. FWE, FDR, permutation or Monte Carlo).*

Models & analysis

n/a	Involved in the study
☐	☐ Functional and/or effective connectivity
☐	☐ Graph analysis
☐	☐ Multivariate modeling or predictive analysis

Functional and/or effective connectivity	*Report the measures of dependence used and the model details (e.g. Pearson correlation, partial correlation, mutual information).*
Graph analysis	*Report the dependent variable and connectivity measure, specifying weighted graph or binarized graph, subject- or group-level, and the global and/or node summaries used (e.g. clustering coefficient, efficiency, etc.).*
Multivariate modeling and predictive analysis	*Specify independent variables, features extraction and dimension reduction, model, training and evaluation metrics.*
