# Supplementary material for: Mitigation of off-target toxicity in CRISPR-Cas9 screens for essential non-coding elements
Source: Nat Commun. 2019 Sep 6;10:4063. doi: 10.1038/s41467-019-11955-7 (PMC6731277; doi:10.1038/s41467-019-11955-7)
Supplement: Supplementary file 3 — Description of Additional Supplementary Files [file 41467_2019_11955_MOESM3_ESM.pdf]

Description of Additional Supplementary Files

Title: Supplementary Data 1

Description: sgRNA libraries used in this study The sgRNA sequences and the sgRNA scores from GuideScan are provided in a separate Excel file.
